# Structural disorder and induced folding within two cereal, ABA stress and ripening (ASR) proteins

**DOI:** 10.1038/s41598-017-15299-4

**Published:** 2017-11-14

**Authors:** Karama Hamdi, Edoardo Salladini, Darragh P. O’Brien, Sébastien Brier, Alexandre Chenal, Ines Yacoubi, Sonia Longhi

**Affiliations:** 1 Laboratoire de Protection et d’Amélioration des Plantes, Centre de Biotechnologie de Sfax (CBS), Sfax, Tunisia; 2 Aix-Marseille Univ, CNRS, Architecture et Fonction des Macromolécules Biologiques (AFMB), UMR 7257 Marseille, France; 3 Institut Pasteur, CNRS UMR 3528, Unité de Biochimie des Interactions Macromoléculaires, Département de Biologie Structurale et Chimie, Paris, France; 4 Institut Pasteur, CNRS USR 2000, Unité de Spectrométrie de Masse Structurale et Protéomique, Paris, France

## Abstract

Abscisic acid (ABA), stress and ripening (ASR) proteins are plant-specific proteins involved in plant response to multiple abiotic stresses. We previously isolated the ASR genes and cDNAs from durum wheat (TtASR1) and barley (HvASR1). Here, we show that HvASR1 and TtASR1 are consistently predicted to be disordered and further confirm this experimentally. Addition of glycerol, which mimics dehydration, triggers a gain of structure in both proteins. Limited proteolysis showed that they are highly sensitive to protease degradation. Addition of 2,2,2-trifluoroethanol (TFE) however, results in a decreased susceptibility to proteolysis that is paralleled by a gain of structure. Mass spectrometry analyses (MS) led to the identification of a protein fragment resistant to proteolysis. Addition of zinc also induces a gain of structure and Hydrogen/Deuterium eXchange-Mass Spectrometry (HDX-MS) allowed identification of the region involved in the disorder-to-order transition. This study is the first reported experimental characterization of HvASR1 and TtASR1 proteins, and paves the way for future studies aimed at unveiling the functional impact of the structural transitions that these proteins undergo in the presence of zinc and at achieving atomic-resolution conformational ensemble description of these two plant intrinsically disordered proteins (IDPs).

## Introduction

Abscisic acid (ABA), stress and ripening (ASR) proteins are a family of plant-specific proteins that have been reported in many species ranging from gymnosperms, (i.e. ginko)^1^, to monocots (i.e. rice and maize)^2,3^, and dicots (i.e. grape)^4^. Despite their broad occurrence in plants, ASR proteins lack orthologues in *Arabidopsis spp*. ASR proteins play a crucial role in plant response to multiple abiotic stresses^5–7^. While some ASR proteins are found exclusively in the nucleus, where they act as transcription factors regulating gene expression during stress response, other ASR proteins are localized in both the nucleus and the cytosol^8^. Beyond this, ASR proteins also act as chaperone-like proteins; for example, tomato ASR1 was found to protect enzymes against freezing or heat denaturation *in vitro*, and similar results were obtained with ASR proteins from plantain and lily^9^.

ASR proteins are small, hydrophilic and highly charged proteins^10^, for which structural data is scarce (for a recent review see^9^). Currently, the best structurally characterized ASR protein is tomato ASR1^11^. Tomato ASR1 is a charged 13-kDa protein, enriched in Gly (7%), Ala, (13%), Glu (15%), His (15%), and Lys (17%)^12^. It is over-expressed under water- and salt-stress conditions, and possesses a zinc-dependent DNA-binding activity^6^. Biophysical methods consistently show that tomato ASR1 is an intrinsically disordered protein (IDP) which folds under desiccation and upon addition of zinc ions^11^. More recently, another ASR protein, namely the product of the MpASR gene from plantain, was also found to be intrinsically disordered^10^. In line with these findings, several studies have classified members of the ASR protein family within the superfamily of Late Embryogenesis Abundant (LEA) proteins. LEA proteins are proteins that accumulate in response to low water availability^13–16^, and most members of this superfamily are either entirely or partially disordered^8^.

In recent years, IDPs have attracted much attention. IDPs lack a well-defined structure in their native state and under physiological conditions^17–21^, and exist as highly dynamic and heterogeneous conformational ensembles in the absence of their partner(s) (for a recent review on IDPs, see^22,23^). IDPs or proteins possessing intrinsically disordered regions (IDRs) are frequently involved in essential processes such as transcriptional and translational activation, chromatin remodeling, and signal transduction. Recent studies have investigated the abundance of intrinsic disorder (ID) in plant proteins^8,24,25^. The inability of plants to move away from a danger has probably been a major driving factor in the development of their stress response. Plants exhibit a high ability to adapt to variable environmental conditions. Taking into consideration that ID serves as a determinant of interactivity, it has been proposed that it may contribute to such plasticity in plants^26^.

We have previously isolated the ASR genes and cDNA from durum wheat (TtASR1) and barley (HvASR1) seedlings under abiotic stress conditions (accession numbers KX660743 and KX660744, respectively). Using computational analyses, we show that these proteins possess the peculiar sequence features of IDPs. We then report their bacterial expression, purification and characterization. Using various complementary biochemical and biophysical methods, we show that both TtASR1 and HvASR1 proteins are disordered. Their spectroscopic and hydrodynamic properties are nevertheless indicative of the presence of some residual secondary and tertiary structure. They were found to undergo a disorder-to-order transition in the presence of glycerol, zinc or 2,2,2-trifluoroethanol (TFE).

## Results

### Disorder predictions and sequence properties of HvASR1 and TtASR1

**Figure 1:**
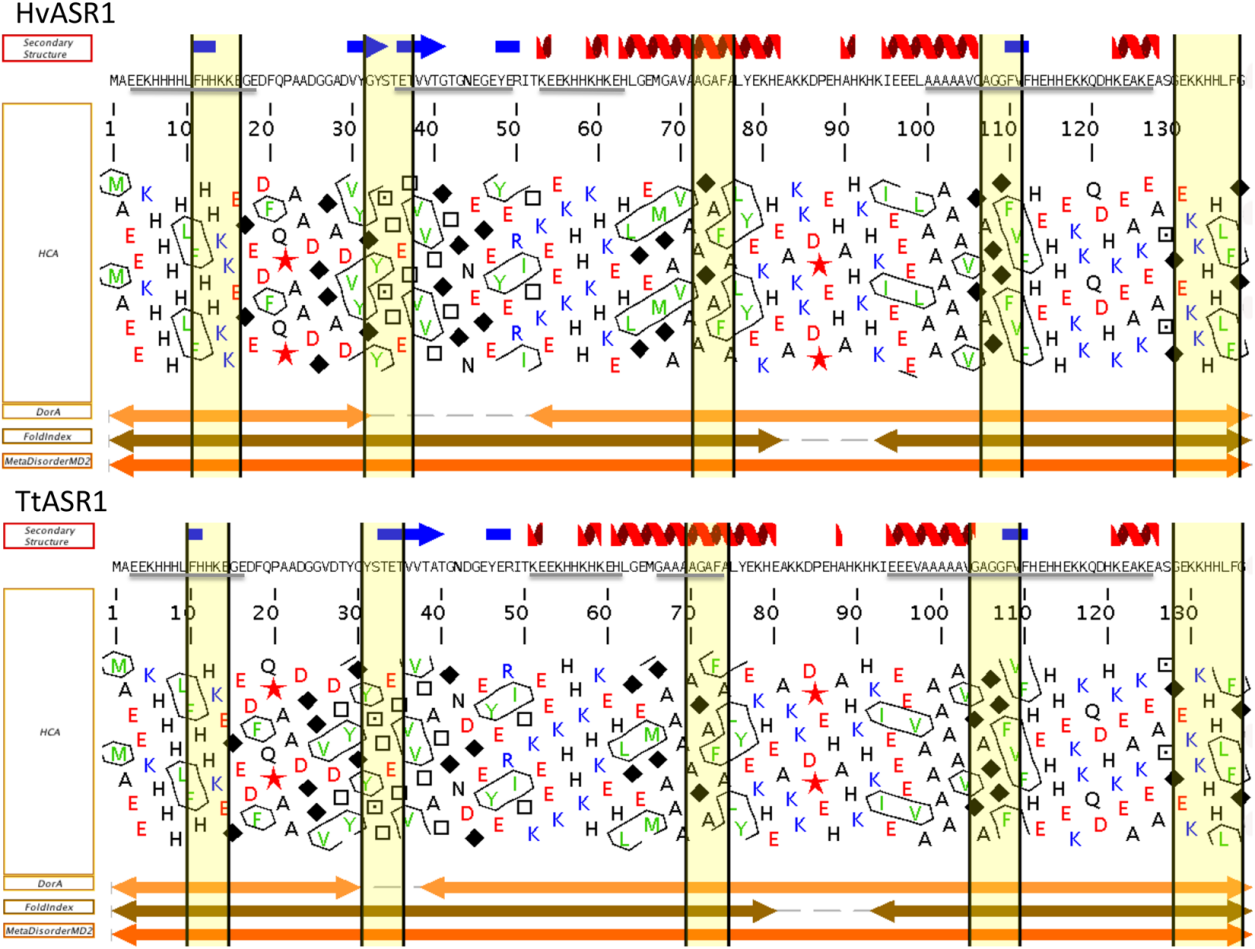
Disorder prediction of HvASR1 and TtASR1. MeDor ouputs of HvASR1 (**A**) and TtASR1 (**B**) proteins. The sequences are represented as single, continuous horizontal lines below the predicted secondary structure elements. Low sequence complexity regions (HvASR1: aa 3–16, 35–49, 53–63, 100–127; TtASR1: aa 3–16, 51–61, 66–74 and 94–125) are shown by a grey bar below the sequence. Below the sequences are shown the HCA plots and the predicted regions of disorder that are represented by bi-directional arrows. MoREs, as predicted by MoRFpred, are highlighted in yellow.

The amino acid sequences of HvASR1 and TtASR1 were analyzed using the metaserver for predicting protein disorder (MeDor) and Genesilico MetaDisorder metaservers (Fig. 1). From hydrophobic cluster analysis (HCA), both proteins appear depleted in hydrophobic clusters, indicative of protein disorder (Fig. 1). Additionally, the two proteins are predicted to be disordered over most of their sequence by DorA and FoldIndex (Supplementary Text 1). In further support of this, both proteins are predicted to be disordered over their entire sequence by MetaDisorderMD2 (Fig. 1), the most accurate disorder predictor according to CASP9^27^. Although HvASR1 and TtASR1 are predominantly disordered, they do posses short regions locally enriched in hydrophobic clusters (Fig. 1). We have previously reported^28–31^, that such regions often correspond to Molecular Recognition Elements (MoREs), i.e. short order-prone regions within IDRs with a propensity to undergo induced folding upon binding to a partner^32^. MoRFpred predicts five such regions (Fig. 1). By comparing the sequence composition of HvASR1 and TtASR1 to that of proteins within the SWISS-PROT database, both proteins were found to be enriched in the most disorder-promoting amino acids (A, G, R, D, H, Q, K, S, E and P) and depleted in order-promoting residues (W, F, Y, I, M, L, V, C and T) (Supplementary Fig. S1)^19,33^. Five amino acids (EAHKG) constitute ~68% of the total TtASR1 and HvASR1 residues (Supplementary Fig. S1). This biased sequence composition is also mirrored by the occurrence of four low sequence complexity regions, as identified by the SEG program^19^ (Fig. 1). According to the scale developed by^34^, Glu (E) is the most disorder-promoting amino acid, and it is also the most abundant in both proteins, representing as much as 16.1% and 16.5% of all residues. Finally, HvASR1 and TtASR1 are also predicted to be disordered by their mean hydrophobicity/mean net charge ratio^18^, as judged from their location in the left-hand side of the RH-plot (Supplementary Fig. S2). Their CH-distance from the boundary hydropathy is very similar (0.061 for HvASR1 and 0.062 for TtASR1), supporting a very similar degree of disorder.

Pappu and colleagues recently indicated that sequence polarity and linear distribution of opposite charges were the primary determinants of the chain dimensions and conformational classes of IDPs^35^. Polar IDPs were found to favor collapsed ensembles in water, despite the absence of hydrophobic groups^36^. To evaluate sequence polarity, we calculated the net charge per residue (NCPR, f_+_ − f_−_)^36,37^, total fraction of charged residues (FCR, f_+_ + f_−_), and linear distribution of opposite charges (κ value)^38^ (Table 1). For both proteins, the NCPR is negative and well below the threshold of 0.2 that discriminates collapsed from extended IDPs^36^, suggesting that they would preferentially populate collapsed states. They belong to the category of weak polyelectrolytes/polyampholytes, where |f_+_ − f_−_| ≤ 0.2 and both f_+_ and f_−_ are small. In addition, they have an FCR value of ~0.3. FCR allows for the discrimination between compact globules and swollen coils, leading to a predictive diagram of states^35^, where region 1 accommodates low-FCR sequences (either weak polyampholytes or polyelectrolytes) that adopt globule or tadpole-like conformations; region 3 houses high-FCR (strong polyampholytes) and low-NCPR sequences that adopt non-globular conformations swollen coil-like conformations (i.e. coil-like and hairpin-like); and region 2 is the boundary between regions 1 and 3, where proteins adopt conformations that likely represent a continuum of possibilities between 1 and 3. ASR1 proteins fall in phase diagram region (PDR) 2^35^ (Supplementary Fig. S2). Furthermore, in region 3 and upper region 2, linear patterning of opposite charges plays a crucial role in defining the chain dimensions of swollen coils. This patterning is quantified by a single parameter, κ, that varies from 0 (when opposite charges are well mixed) to 1 (when opposite charges are segregated). The average chain dimension of IDPs (radius of gyration, Rg) shows strong anti-correlation with the κ value^38^. The very low κ value of the ASR1 proteins suggests that these proteins adopt a rather extended and swollen, coil-like conformation.

**Table 1:** Sequence properties of ASR1 proteins.

Protein	Hydropathy	pI	N	f_+_	f_−_	NCPR	FCR	*k*	PDR
HvASR1	3.37	6.10	138	0.136	0.201	−0.065	0.34	0.101	2
TtASR1	3.40	5.99	136	0.131	0.204	−0.074	0.33	0.099	2

Protein sequences examined here include the initial methionine and no vector-encoded, non-native residues. N: residue number. f_+_: fraction of positively charged residues. f_−_: fraction of negatively charged residues. FCR: fraction of charged residues (f_+_ + f_−_). NCPR: net charge per residue, value of the difference between the fraction of positively charged and negatively positively residues, (f_+_ − f_−_). The *k-*value was computed as described^38^. PDR: phase diagram region as defined in^38^.

In conclusion, all of the *in silico* analyses consistently converge to predict that HvASR1 and TtASR1 are members of the family of IDPs that adopt a swollen coil-like conformation.

### Expression and purification of HvASR1 and TtASR1 proteins

**Figure 2:**
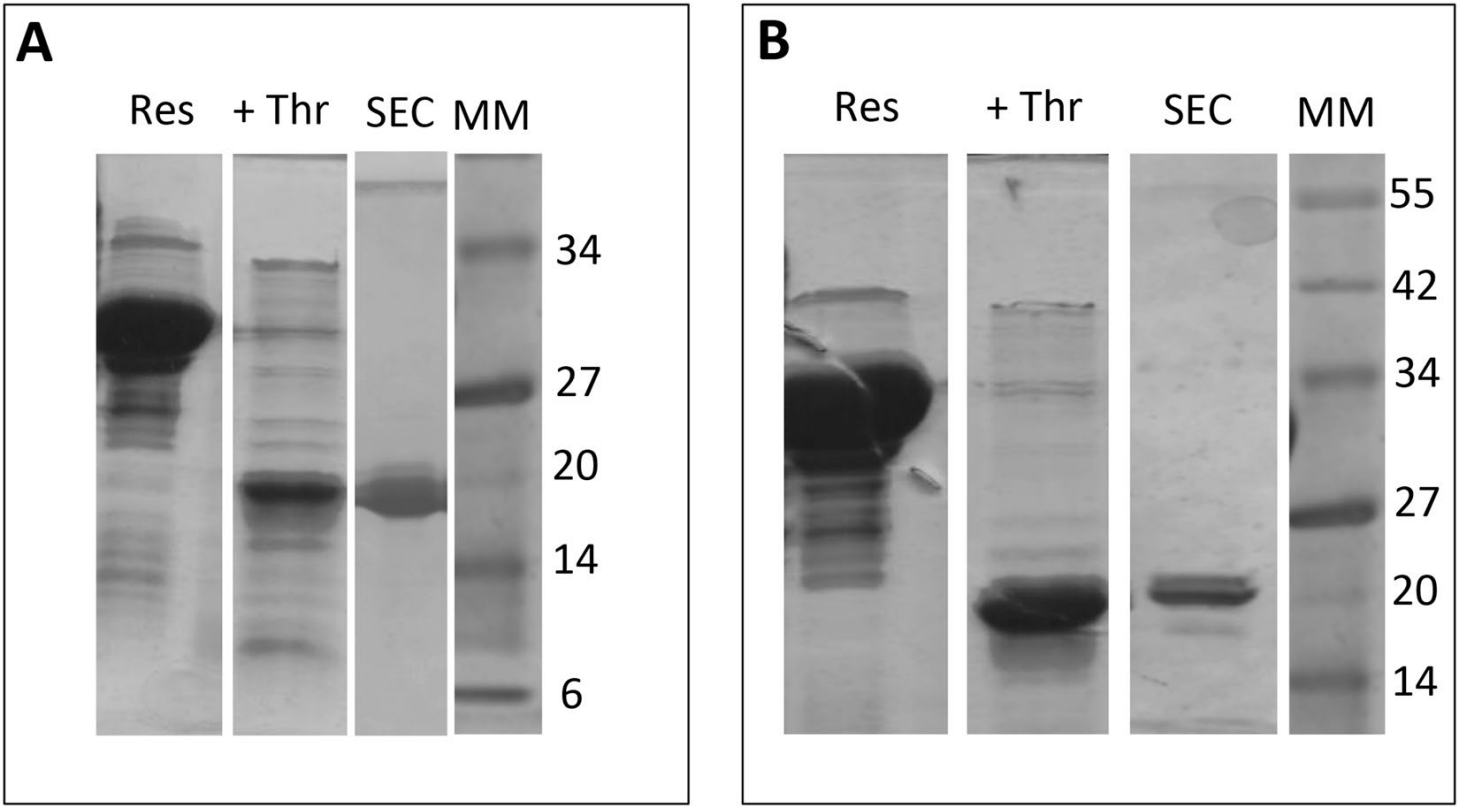
Purification of HvASR1 (**A**) and TtASR1 (**B**) proteins from *E*. *coli*. Coomassie blue staining of SDS-PAGE showing the affinity chromatography resin after extensive washing (Res), the released protein after thrombin cleavage (+Thrombin) and the final purified product as obtained after SEC (SEC). MM: molecular mass markers.

**Figure 3:**
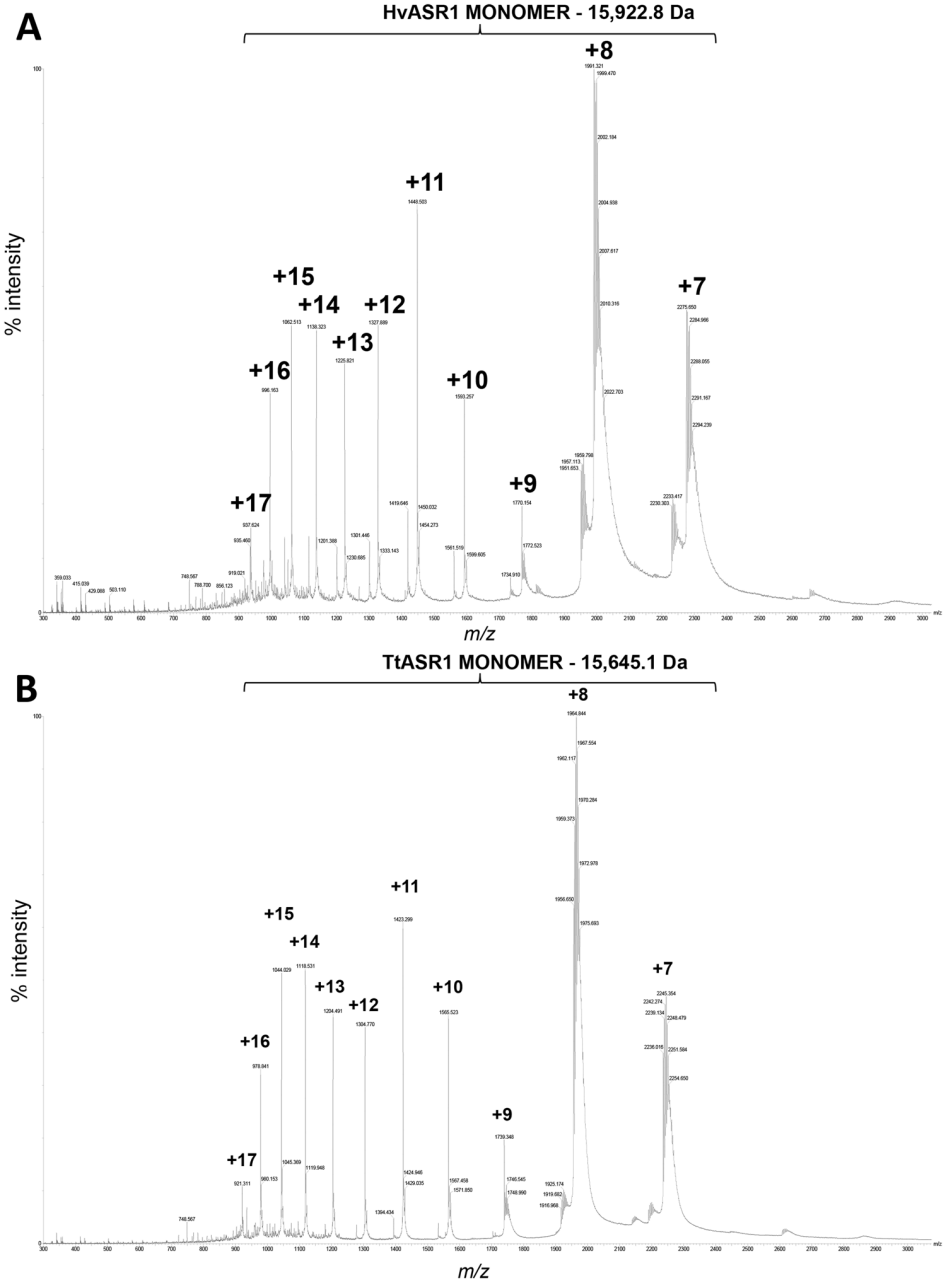
Native ESI-MS spectra of HvASR1 (**A**) and TtASR1 (**B**) in 250 mM ammonium acetate, pH 8 (Synapt G2-Si mass spectrometer, Waters).

To experimentally assess the disordered nature of HvASR1 and TtASR1, we cloned the cDNAs encoding full-length ASR1 proteins into the pGEX4-T1 expression vector that allows the inducible expression in *E*. *coli* of N-terminally glutathione S transferase (GST) tagged proteins. The tagged proteins were purified by affinity chromatography, followed by thrombin cleavage to remove the GST tag and size-exclusion chromatography (SEC) (Fig. 2). Both HvASR1 and TtASR1 exhibit an abnormally slow migration in SDS-PAGE, with an apparent molecular mass (MM) comprised between 20 and 25 kDa (expected MM ~16 kDa) (Fig. 2). MALDI-TOF and native electrospray ionization (ESI) mass spectrometry (MS) analyses yielded the exact molecular mass expected for both proteins (data not shown and Fig. 3). This aberrant migration during electrophoresis is a hallmark of IDPs and is often due to their typically high content of acidic and negatively charged residues, which results in a lower binding of Sodium dodecyl sulfate (SDS) than usual^21^. As a result, their apparent MM is often 1.2–1.8 times higher than that which is calculated from sequence data or measured by MS. Furthermore, we have previously reported that the degree of protein extension in solution is an additional parameter affecting the electrophoretic mobility of IDPs^39^. The aberrant electrophoretic migration of HvASR1 and TtASR1 proteins constitutes the first experimental hint of their disordered nature.

### Hydrodynamic properties of HvASR1 and TtASR1 proteins from SEC

To investigate the hydrodynamic properties of HvASR1 and TtASR1 we used analytical SEC. The two proteins were eluted from the gel filtration column as sharp peaks (data not shown), with a similar elution volume. The elution profiles were similar, irrespective of whether sodium phosphate or Tris/HCl buffer was used, and regardless of the NaCl concentration. The Stokes radius (R_S_) was estimated to be 24.7 ± 2 Å for HvASR1, and 25.2 ± 2 Å for TtASR1 (see Table 2). These very high Stokes radius (R_S_) values can’t be ascribed to protein aggregation, since they are independent from protein concentration (data not shown). Different protein conformational classes have characteristic hydrodynamic dimensions and molecular mass correlations^40^. IDPs have larger hydrodynamic dimensions compared to typical native globular proteins^40^. By comparing the experimentally determined Stokes radius (R_S_^obs^) of HvASR1 and TtASR1 with the Stokes radii expected for various conformational states (native, molten globule, pre-molten globule, and denaturant-unfolded), the two proteins were found to have an R_S_ higher than the value expected for a monomeric, natively folded form (~20 Å) (Table 2). The experimentally observed values of the R_S_ are very close to those expected either for a premolten globule (PMG) form (i.e. an extended conformation possessing some residual structure^40^) (~27.3 Å), or for a molten globule (MG) form (~22.3 Å), or for a folded dimer (~25.5 Å) (see ratios between R_S_^obs^ and R_S_^PMG^ or R_S_^MG^ or R_S_^DimNF^ in Table 2). These results suggest that HvASR1 and TtASR1 proteins are either folded dimers or IDPs adopting a MG or PMG conformation.

**Table 2:** Stokes radii (R_S_^obs^, Å) as obtained by SEC and expected values for the various conformational states.

Protein	Mass (Da)	R_S_^obs^	R_S_^NF^	R_S_^MG^	R_S_^PMG^	R_S_^U^	R_S_^IDP^	R_S_^Dim NF^	R_S_^obs^/R_S_^MG^	R_S_^obs^/R_S_^PMG^	R_S_^obs^/R_S_^U^	R_S_^obs^/R_S_^IDP^	R_S_^obs^/R_S_^Dim NF^	CI
HvASR1	15922	24.7	19.8	22.4	27.4	34.7	31.2	25.5	1.10	0.90	0.71	0.79	0.97	0.67
TtASR1	15645	25.2	19.7	22.3	27.2	34.4	31.0	25.5	1.13	0.92	0.73	0.81	0.99	0.62

R_S_^NF^: R_S_ expected for a natively folded form; R_S_^MG^: R_S_ expected for a molten globule (MG); R_S_^PMG^: R_S_ expected for a premolten globule (PMG); R_S_^U^: R_S_ expected for a fully unfolded form; R_S_^IDP^: R_S_ expected for an IDP based on the simple power-law model; R_S_^Dim NF^: R_S_ expected for dimeric, folded form. M: molecular mass calculated from the amino acid sequence of the recombinant protein. CI: compaction index.

### Native electrospray ionization mass spectrometry (ESI-MS) analysis of HvASR1 and TtASR1 proteins

To determine whether the ASR1 proteins are prevalently unfolded monomers or folded dimers, we carried out native electrospray ionization MS (ESI-MS) analysis of the HvASR1 and TtASR1 proteins. To ascertain the suitability of the approach to detect possibly occurring oligomeric species in a protein sample, we first carried out control experiments using alcohol dehydrogenase (ADH, a tetrameric protein)^41^. Results show that under the conditions herein used, ADH conserves its tetrameric organization (Supplementary Fig. S3). Results show that both TtASR1 and HvASR1 proteins are present as monomers in the gas phase (Fig. 3). No *m/z* values corresponding to dimeric forms of each were detected thus confirming that the large R_S_ observed in SEC studies reflects a predominantly unfolded monomeric species. The multiple and high charge states observed (up to +17) confirm the intrinsically disordered nature of the proteins.

### Differential scanning fluorimetry of HvASR1 and TtASR1 proteins

The conformation of HvASR1 and TtASR1 was further explored by differential scanning fluorimetry (DSF). This method is used to monitor thermal transitions of proteins in the presence of a fluorescent dye that is highly fluorescent in non-polar environments, such as the hydrophobic pockets of (partly) unfolded proteins, and which is quenched in aqueous solutions and/or in the presence of native proteins (Supplementary Text 2)^42^. As shown in Supplementary Fig. S4, the experimentally observed profiles for HvASR1 and TtASR1 are consistent with lack of a stable 3D structure, as judged from their rather high basal fluorescence at 20 °C and from the flatness of their profile. These results thus confirm their disordered nature and advocate for a PMG rather than a MG conformation.

### Conformational properties of HvASR1 and TtASR1 proteins from small angle X-ray scattering (SAXS) studies

**Figure 4:**
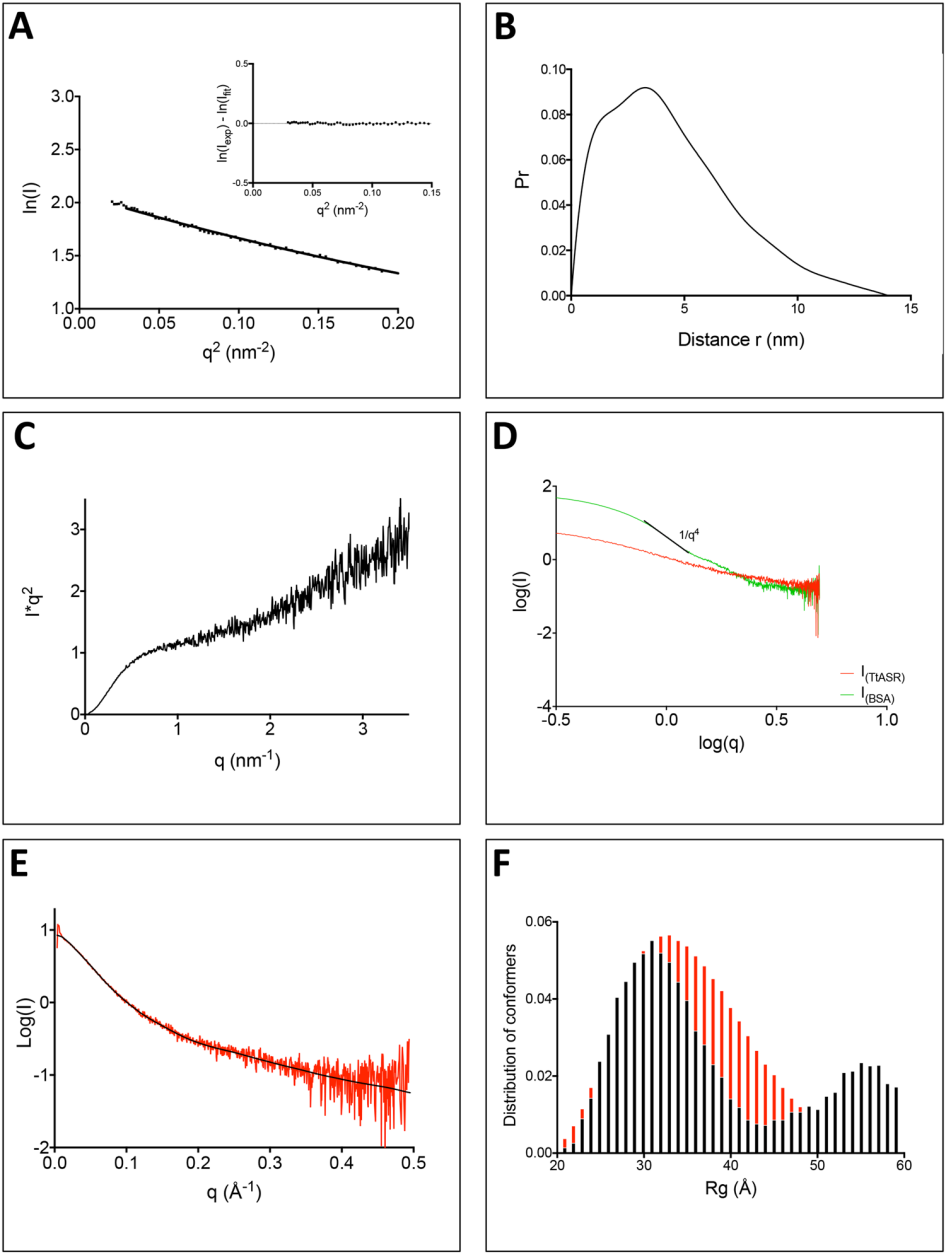
SAXS studies of TtASR1. (**A**) Representation of the Guinier plot for the protein at 3.0 g/L. Inset: residuals. (**B**) Pair distance distribution, P(r), function of the data for the 3.0 g/L concentration. (**C**) Kratky plot of the SAXS data obtained at 1.5 g/L. (**D**) Log-log representation of TtASR1 and BSA SAXS patterns. (**E**) Experimental scattering curve of the protein at 3.0 g/L (red) and GAJOE fit (black). (**F**) Distribution of Rg of the ensemble of randomly generated conformers by Flexible-Meccano without constraints (red) and of the sub-ensemble of selected conformers using GAJOE (black).

Small-angle X-ray scattering (SAXS) is well suited to study flexible, low compactness or even extended macromolecules in solution^43,44^. The SAXS curves and Guinier plots obtained at different protein concentrations are independent of protein concentration, indicating the absence of significant aggregation (data not shown). Each curve can be approximated by a straight line in the Guinier region (qR_g_ < 1.0). The slope gives the value of the radius of gyration, R_g_, while the intercept of the straight line gives the I(0), which is proportional to the molecular mass of the scatterer. Guinier analysis in the low q region gave an R_g_ of 34.6 ± 0.6 Å for HvASR1 and 35.5 ± 0.3 Å for TtASR1 at the highest protein concentration (Supplementary Fig. S5 and Fig. 4A and Table 3). Very similar values were obtained at lower concentrations (Table 4), and in good agreement with the values (35.7 ± 0.2 for HvASR1 and 35.8 ± 0.5 for TtASR1) determined from the pair distance distribution function P(r) (Supplementary Fig. S5 and Fig. 4B and Table 3). The molecular mass determined from the extrapolated scattering intensity at zero angle I(0) is 12.8 kDa for HvASR1 and 13.0 kDa for TtASR1. These values are close, although a bit smaller, to the values expected for a monomeric form of the two proteins (Table 3).

**Table 3:** SAXS data collection and scattering-derived structural parameters (at the highest concentration) for HvASR1 and TtASR1.

	HvASR1	TtASR1
**Data-collection parameters**		
Detector	Pilatus (1 M)	Pilatus (1 M)
Beam geometry	Bending magnet (BM29)	Bending magnet (BM29)
Wavelength (Å)	0.992	0.992
*q* Range (Å^−1^)	0.028–4.525	0.028–4.525
Exposure time (s)	1	1
Concentration range (g/L)	1–1.5	1–3
Temperature (°C)	20	20
**Structural parameters**		
I(0) (cm^−1^) (from Guinier)	12.7 ± 0.8	12.5 ± 0.5
Rg (nm) (from P(r))	3.57 ± 0.02	3.58 ± 0.05
Rg (nm) (from Guinier)	3.46 ± 0.06	3.55 ± 0.03
Dmax (nm)	11.9	12.3
**Molecular-mass determination** (**kDa**)		
Molecular mass (MM) (from I(0))	12.8	13
Calculated MM from sequence	15.9	15.7
**Software employed**		
Primary data reduction	PRIMUS	PRIMUS
Data processing	GNOM	GNOM
Validation of structural models	CRYSOL/GAJOE	CRYSOL/GAJOE

**Table 4:** R_g_ (from Guinier) and D_MAX_ for HvASR1 and TtASR1 at the various protein concentrations.

Protein concentration (g/L)	Rg (nm) (Guinier)	Dmax (nm)
HvASR1 1.0	3.51 ± 0.09	13.47
HvASR1 1.5	3.46 ± 0.06	11.90
TtASR1 1.0	3.31 ± 0.08	14.87
TtASR1 1.5	3.49 ± 0.06	11.75
TtASR1 2.0	3.58 ± 0.02	12.17
TtASR1 3.0	3.55 ± 0.03	12.31

The R_g_ value obtained for the ASR1 proteins (~35 Å) is 2.25 times higher than that expected for globular, fully unfolded proteins and IDPs of the same size (~15.5 Å) (see Eq. 10 in Methods)^45^ On the other hand, the expected value for a fully unfolded form is ~110 Å (see Eq. 12 in Methods). The R_g_ values expected for IDPs of the same length as the ASR1 proteins, as calculated using Flory’s power law and parameters based on an IDP pool^46^, are 33.9 Å for HvASR1 and 33.6 Å for TtASR1 (see Eq. 10 in Methods). The experimental R_g_ values are therefore in very good agreement with the expected R_g_ values using parameters inferred from IDPs, while they are three times smaller that those expected for fully unfolded forms. Denaturing conditions are indeed known to lead to excluded volume effects around the polypeptide chain, thereby resulting in higher values of R_g_, compared to IDPs. The experimentally observed R_g_ values are ~1.7 times larger than those expected for globular proteins (~19 Å), with an R_s_ equal to that experimentally observed for the ASR1 proteins (~25 Å) (see Eq. 13 in Methods), thus suggesting an extended shape. They are both much higher than the expected value for a sphere (~12.8 Å), with an R_s_ of ~25 Å, as determined from the volume of a sphere with either 141 or 143 residues (see Methods). The strong discrepancy between the experimentally observed R_g_ values and those expected for a globular/spherical form, together with the good agreement with those expected for IDPs, indicate that the ASR1 proteins are disordered. The distribution of internal distances, as inferred from the scattering curves obtained at the highest protein concentration, yielded a maximal internal dimension D_max_ of 119 Å for HvASR1 and of 123 Å for TtASR1 (Supplementary Fig. S5 and Fig. 4B). This large D_max_ also indicates that the proteins are extended.

The Kratky plots of the two ASR1 proteins (Supplementary Fig. S5 and Fig. 4C) have plateaus >1.0 nm^−1^. The absence of a maximum clearly indicates that the proteins are not globular and do not possess a tightly packed core (Supplementary Text 3). In addition, from the log-log representation of the scattering curves, no region with a s^−4^ dependence above 0.1 Å^−1^ was observed (Supplementary Fig. S5 and Fig. 4D), a feature indicative of the absence of a clear boundary between the solvent and the protein surface. By constrast, the curve of BSA exhibits a region with a s^−4^ dependence characteristic of the presence of a sharp interface between the solvent and a compact, well-folded domain, as stated in Porod’s law^47^.

We then assessed the distribution of conformations of the ASR1 proteins in solution. For each protein, a pool of 10,000 conformations was randomly generated using Flexible-Meccano. From this initial ensemble, a sub-ensemble of conformers that collectively reproduces the experimental SAXS data and represents the distribution of structures adopted by the protein in solution was selected. The average SAXS scattering curves back-calculated from the selected sub-ensembles reproduce correctly the experimental curves (χ^2^ of 0.70 for HvASR1 and 0.78 for TtASR1) (Supplementary Fig. S5 and Fig. 4E). The R_g_ distribution of the initial ensembles is broad and symmetrical with values extending from 20 to 60 Å nm with a maximum frequency near 33 Å (Supplementary Fig. S5 and Fig. 4E, red bars). For both proteins, the R_g_ distribution of the selected sub-ensemble is a bit wider and bimodal, i.e. it displays two peaks, centered on 33 and 60 Å, suggesting the presence of two distinct sub-populations of conformers (Supplementary Fig. S5 and Fig. 4F, black bars). This implies that the scattering curves of the two ASR1 proteins do not reflect a randomly distributed ensemble of conformations. Note that successive and independent selections by GAJOE yielded similar ensembles of conformers, indicating that the distribution of the selected sub-ensemble was reproducible (data not shown).

Altogether, these analyses consistently confirm the disordered nature of the ASR1 proteins.

### Circular dichroism (CD) studies of HvASR1 and TtASR1 proteins

**Figure 5:**
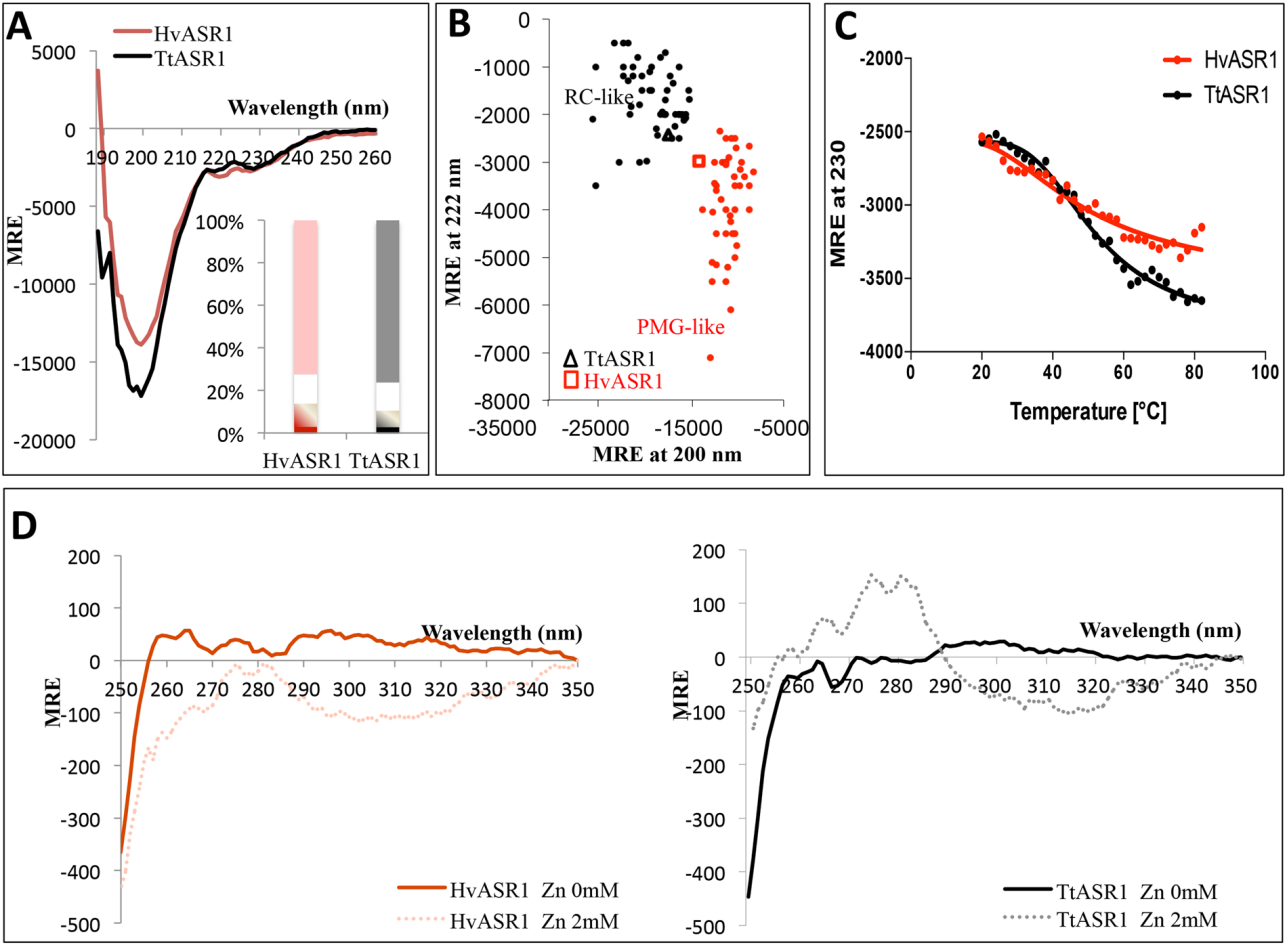
Analysis of HvASR1 and TtASR1 by circular dichroism. (**A**) Far-UV CD spectra of HvASR1 and TtASR1 at 0.1 mg/mL in 10 mM sodium phosphate pH 7 at 20 °C. Data are representative of one out of three independent measurements. The inset shows the secondary structure content of the two proteins, as derived using CDSSTR. Grey or light red: unordered; white: turns; black or red: helix; hatched: strand. (**B**) Plot of the molar residue ellipticity (MRE) at 222 nm and at 200 nm of a set of well-characterized unfolded, random coil-like (RC-like) or premolten globule-like (PMG-like) proteins (from^40^). The position in the plot of HvASR1 and TtASR1 is highlighted by a square and a triangle, respectively. (**C**) Molar residue ellipticity (MRE) at 230 nm of HvASR1 and TtASR1 as a function of the temperature. The experimental points in the 20–80 °C range were fitted to a sigmoidal curve. Protein and buffer concentrations were the same as described in (**A**). (**D**) Near-UV CD spectra of HvASR1 and TtASR1 at 1 mg/mL in 10 mM sodium phosphate pH 7 in the absence or presence of 2 mM ZnSO_4_ at 20 °C. Data are representative of one out of two independent acquisitions.

To estimate the secondary structure content of HvASR1 and TtASR1, we recorded their circular dichroism (CD) spectra in the far ultraviolet (UV) region. Under native conditions and neutral pH, both proteins present spectra typical of proteins lacking any stable organized secondary structure, as judged from their large negative ellipticity at 200 nm, low amplitude in the 210–230 nm region, and low ellipticity at 190 nm (Fig. 5A). Spectral deconvolution revealed a high content (>60%) of unordered structure in both ASR1 proteins (see insets in Fig. 5A).

It has been previously noticed that IDPs can be subdivided in PMG-like and Random Coil-like (RC-like) forms as a function of their ellipticity values at 200 and 222 nm^40^. As shown in Fig. 5B, HvASR1 and TtASR1 fall in the RC-like and PMG-like region, respectively, with both being located close to the boundary between the two classes. This indicates that TtASR1 is slightly more extended than HvASR1, a finding in agreement with SEC data that highlighted a slightly smaller compaction index (CI) for TtASR1 (see Table 2).

We also monitored the ellipticity at 230 nm as a function of temperature (Fig. 5C). In both cases, increasing the temperature globally results in an increase in the ellipticity (Fig. 5C). While the profile obtained for TtASR1 could be satisfactorily fitted to a sigmoidal curve (with a calculated inflection point of folding of 51 ± 8 °C), the quality of the fit for HvASR1 was poor, leading to a much less reliable estimation of the folding temperature (61 °C) (Fig. 5C). Regardless of these subtle differences, the profiles indicated that both ASR1 proteins undergo temperature-induced folding, as already observed for other IDPs^48,49^. This heat-induced folding is reversible, with the spectra reverting to their initial appearance after re-cooling at room temperature (data not shown). To rule out any possible temperature-induced aggregation, we monitored the HT voltage associated with the transmission of light through the proteins in the CD spectropolarimeter during heating of the proteins. We observed no significant changes in the HT voltage associated with heating, indicating a lack of aggregation (data not shown).

We also recorded the CD spectra of HvASR1 and TtASR1 in the near ultraviolet region (250–350 nm). Near-UV CD spectra reflect the environment of the aromatic amino acid side chains and provide information about the tertiary structure of proteins^50^. The near-UV CD spectra of both proteins are characterized by a weak intensity, and by the presence of a broad negative band (Fig. 5D), indicating that unlike typical globular proteins, these two proteins lack a hydrophobic core containing oriented aromatic residues, i.e. they lack a stable tertiary structure^51^.

### Folding induced by glycerol

**Figure 6:**
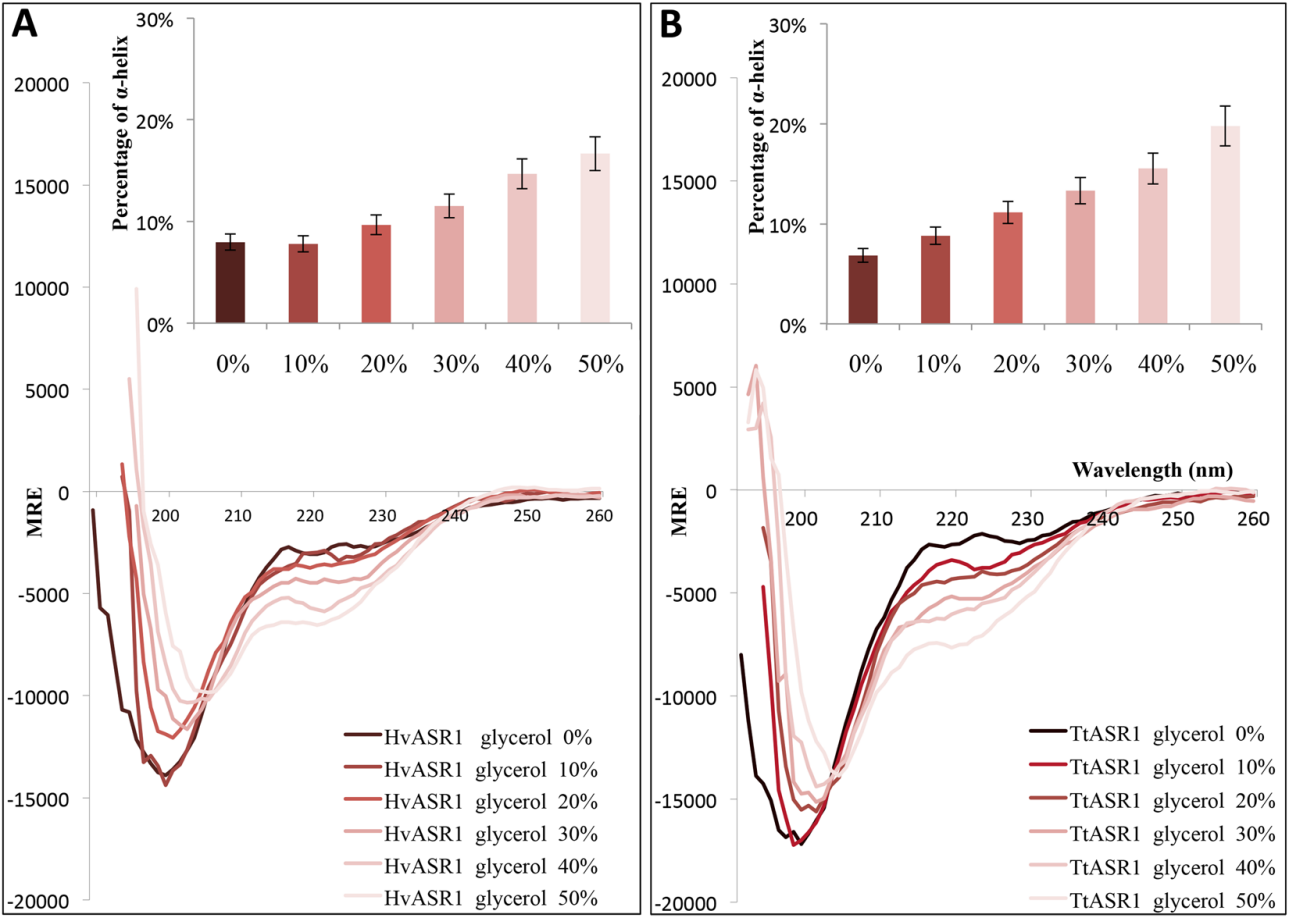
Far-UV CD spectra of HvASR1 (**A**) and TtASR1 (**B**) in the presence of increasing concentrations of glycerol at 20 °C. Proteins were at 0.1 mg/mL in 10 mM sodium phosphate pH 7. Data are shown to the point up to which the dyna voltage was in the permissible range. Data are representative of one out of two independent acquisitions. The insets show the α-helical content as derived as described in Methods (see Eq. 14). MRE: molar residue ellipticity.

As desiccation has been previously reported to induce folding of tomato ASR1^11^, as well as of a number of LEA proteins^52,53^, we sought to assess whether HvASR1 and TtASR1 also undergo some degree of folding under such conditions. In this regard, we recorded their far-UV CD spectra in the presence of increasing concentrations of glycerol, a condition that mimics dehydration. As shown in Fig. 6, addition of increasing glycerol concentrations triggers a gradual increase in the α-helical content. Note that for both proteins, the spectra display an isodichroic point around 205 nm, a behavior indicative of a two-state transition.

To ascertain whether the gain of structure observed in the presence of glycerol arises from dehydration or from crowding effects (i.e. increased excluded volume effects), we also recorded the far-UV CD spectra of both ASR1 proteins in the presence of 30% of sucrose. The addition of sucrose does not induce a pronounced folding in the two ASR1 proteins and triggers only a modest increase in the α-helical content of HvASR1 (Supplementary Fig. S6), that is not mirrored in TtASR1 (Supplementary Fig. S6). In the latter however, the overall content in disorder slightly decreases, as judged from the decrease in the amplitude of the negative peak at 200 nm upon addition of sucrose. In neither case, the structural transition is comparable to that observed with glycerol, advocating for a mechanism where the protein probably folds not because of excluded volume effects, but rather because hydrogen bonds with water molecules are replaced by intramolecular ones.

### Folding induced by TFE

**Figure 7:**
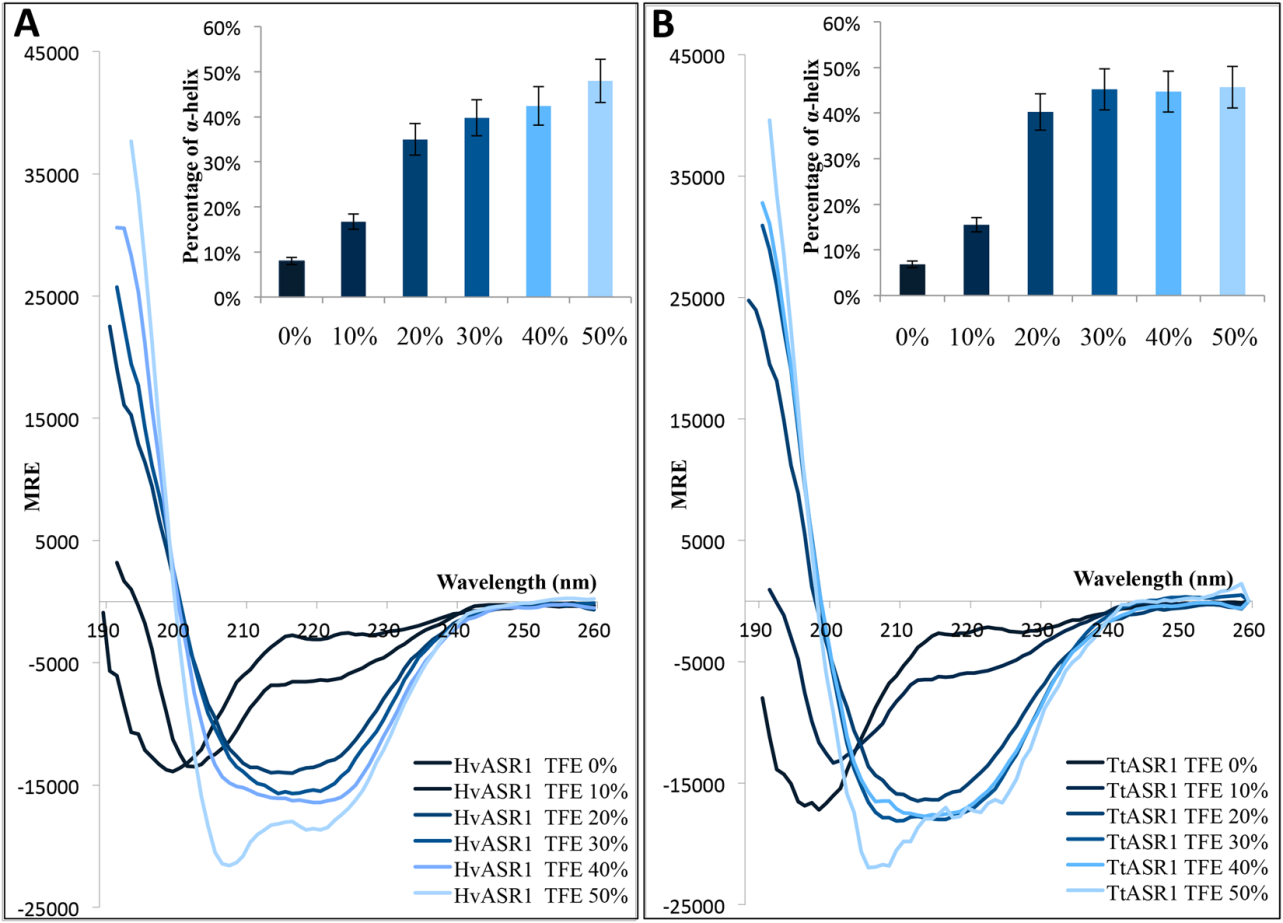
Far-UV CD spectra of HvASR1 (**A**) and TtASR1 (**B**) in the absence or presence of increasing TFE concentrations at 20 °C. Proteins were at 0.1 mg/mL in 10 mM sodium phosphate pH 7. Data are shown to the point up to which the dyna voltage was in the permissible range. Data are representative of one out of two independent acquisitions. The insets show the α-helical content as derived as described in Methods (see Eq. 14). MRE: molar residue ellipticity.

Many IDPs undergo some degree of folding upon binding to their partners/ligands^23^. To further study the potential of HvASR1 and TtASR1 to adopt a stable regular secondary structure, their CD spectra were recorded in the presence of increasing concentrations of TFE (Fig. 7). TFE is a secondary structure stabilizer that mimics the hydrophobic conditions that proteins experience when binding hydrophobic patches in their targets, and is commonly used to probe hidden structural propensities of IDPs (Supplementary Text 4)^54–56^. In the presence of TFE, both HvASR1 and TtASR1 show a notable increase in ordered structure. Their α-helicity increases with increasing TFE concentrations (see inset in Fig. 7). In the presence of 50% of TFE, the spectra exhibit a clear α-helical character, as judged from the appearance of the characteristic double minima at 208 and 222 nm (Fig. 7). Note that for both proteins, no isodichroic point is discernable, arguing for a more than two-state TFE-induced structural transition.

### Protease sensitivity in the absence and presence of TFE and mass spectrometry analyses

**Figure 8:**
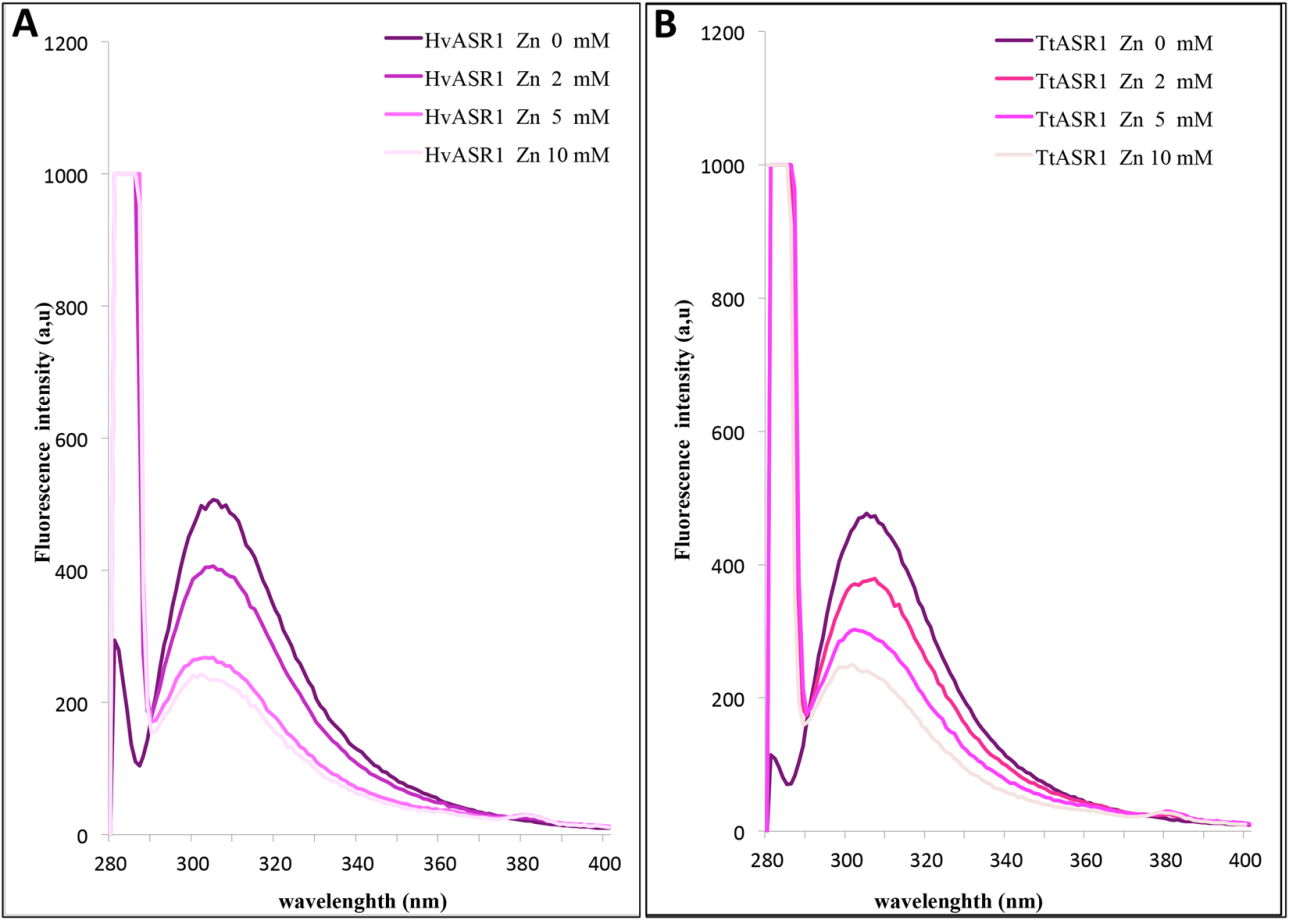
SDS–PAGE analysis of limited digestion of HvASR1 and TtASR1 by thermolysin both in the presence and absence of 15% TFE at 25 °C. The extent of thermolysin digestion of the purified proteins at different time intervals (0, 1, 3, 6 and 24 h) in the presence of 15% TFE is shown. The extent of digestion of the two proteins, as observed after 1 and 6 h in the absence of TFE is also shown. The black arrow shows undigested ASR1 proteins (1) and the red arrow a fragment resistant to proteolysis (2). M: molecular mass markers. The amino acid sequence of the proteins is shown and the region corresponding to the resistant fragment is shown in red. The underlined region corresponds to the region, shared by both ASR1 proteins, that becomes more structured in the presence of zinc (see also Fig. 11).

We also investigated HvASR1 and TtASR1 by limited proteolysis either in the presence or absence of TFE. Limited proteolysis is widely used to identify folded domains within (modular) proteins^57^. Proteolytic cleavage sites within unfolded regions are cut first, with more structured regions remaining undigested^57^. A corollary of this is that IDPs are hypersensitive to proteolysis^21,58^. For digestion, we used thermolysin, a TFE-resistant enzyme that has broad substrate specificity. The latter property allows the identification of cleavage sites solely on the basis of their location in flexible/exposed regions. HvASR1 and TtASR1 were submitted to limited thermolysin digestion either in the presence or absence of 15% TFE, i.e. the minimum concentration to obtain a fragment resistant to proteolysis (Fig. 8). In the absence of TFE, the two proteins are readily degraded after an incubation as short as one hour, and entirely digested after 6 hours (Fig. 8), a behavior that is consistent with an overall high solvent accessibility and disordered nature. Conversely, they show greater resistance to digestion in the presence of 15% TFE (Fig. 8). For both proteins, a few resistant fragments could be detected, of which one was clearly more resistant to digestion than the others, even after an incubation period as long as 24 hours (Fig. 8, arrow 2). Mass spectrometry (MS) analysis allowed the resistant fragment of HvASR1 and TtASR1 to be mapped to the regions encompassing residues 99–122 and 98–118 of both proteins, respectively. Notably, this fragment covers a predicted α-helix and MoRE in each (see Fig. 1). It is therefore reasonable to assume that under these conditions, this fragment is folded as an α-helix, preventing its cleavage and digestion by thermolysin. This region may therefore represent a secondary structure element that is involved in the disorder-to-order transition that the proteins may undergo upon binding their physiological partner(s).

### Effect of zinc

As tomato ASR1 was previously shown to bind zinc ions and undergo Zn-induced folding^11,59^, we checked whether HvASR1 and TtASR1 retain this ability. Binding experiments to a sepharose resin, previously equilibrated with ZnSO_4_, unveiled that the two proteins are retained on the resin after extensive washing (Supplementary Fig. S7). The possibility that the proteins could stick to the resin itself was checked and ruled out (Supplementary Fig. S7). Concomitantly, these experiments also unveiled that the two proteins are equally able to bind to a resin previously equilibrated with NiSO_4_ (Supplementary Fig. S7). Incidentally, these findings also have a practical interest, as they point to a behavior that could be exploited for purification purposes.

**Figure 9:**
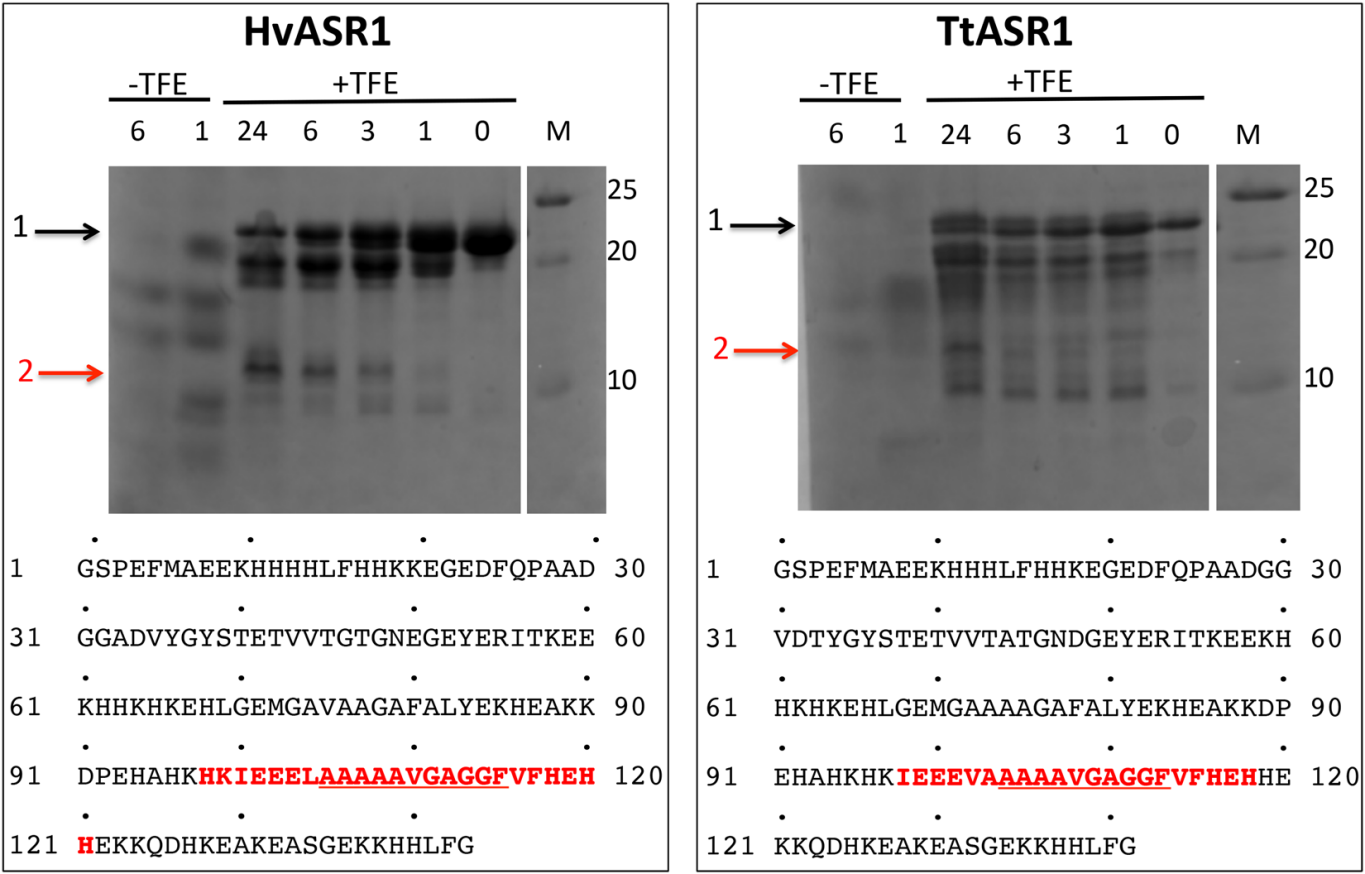
Fluorescence emission spectra of HvASR1 (**A**) and TtASR1 (**B**) at 20 °C. Proteins were at 1 μM in 10 mM sodium phosphate buffer pH7 supplemented with various concentrations of ZnSO_4_.

We next assessed the impact of zinc ions on ASR1 structure. We first recorded the fluorescence emission spectra of the proteins either in the presence or absence of increasing zinc concentrations (Fig. 9). As expected for Tyr-containing proteins, both ASR1 proteins exhibit a maximum close to 303 nm. Interestingly, the intensity of the emission peak is gradually quenched with increasing zinc concentration, reflecting a decrease in the solvent exposure of Tyr residues. This behavior is indicative of a zinc-induced gain of tertiary structure.

Near-UV CD studies further support this conclusion (Supplementary Text 5). For both proteins, the addition of 2 mM ZnSO_4_ results in a spectral modification, consistent with the gain of some tertiary structure (Fig. 5D). In particular, a peak between 275 and 282 nm, which corresponds to Tyr residues^60^, becomes discernible, reflecting a conformational change in the environment of Tyr residues (Fig. 5D). Surprisingly however, the near-UV CD spectra obtained in the presence of zinc differ for the two proteins, a finding that cannot be easily rationalized, as the two proteins have the same number of Tyr (and Phe) residues whose location in the sequence is also conserved.

**Figure 10:**
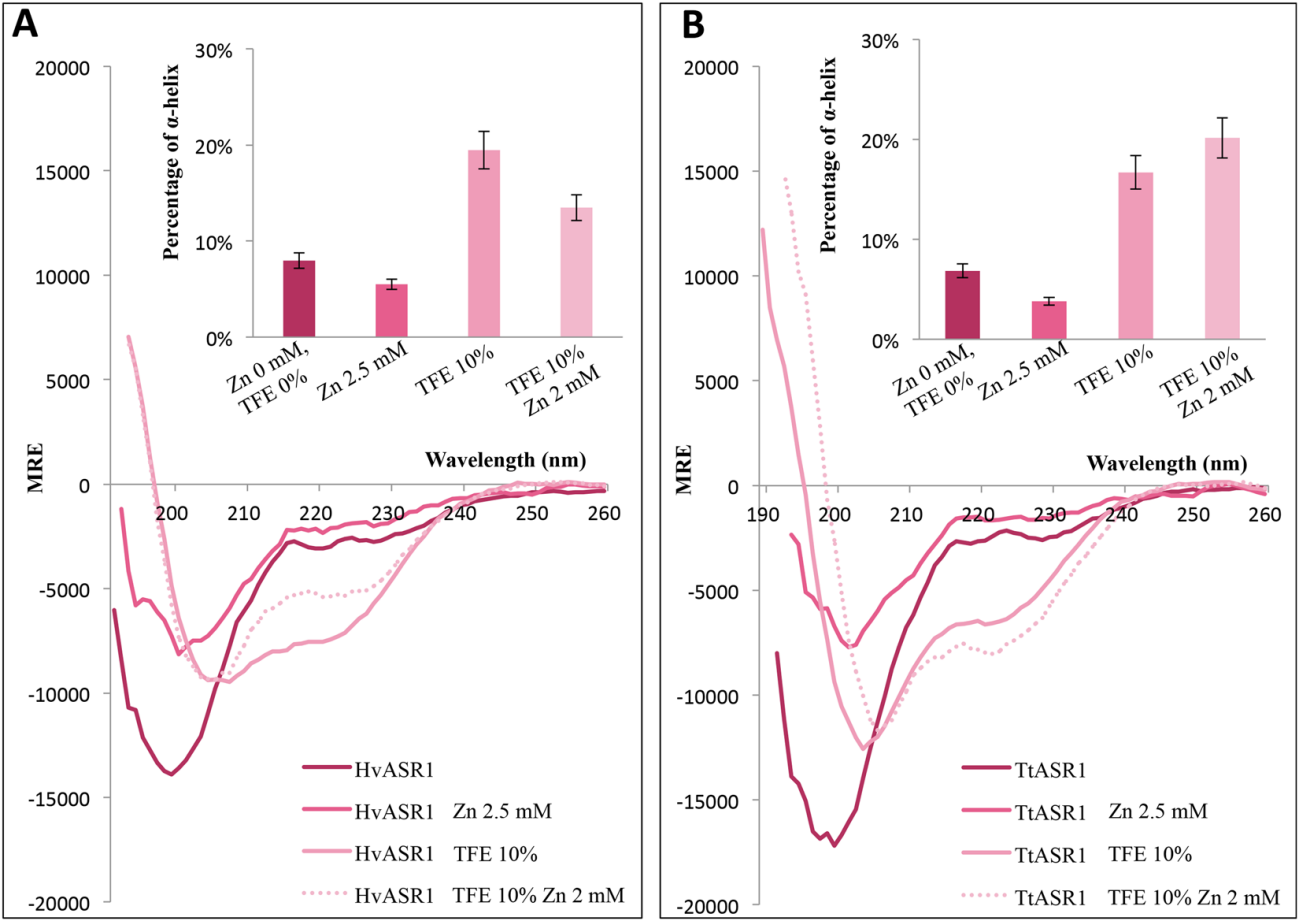
Far-UV CD spectra of HvASR1 (**A**) and TtASR1 (**B**) in the presence or absence of either 2.5 mM ZnSO_4_, or 10% TFE or 10% TFE + 2 mM ZnSO_4_ at 20 °C. Proteins were at 0.1 mg/mL in 10 mM sodium phosphate pH 7. Data is shown to the point at which the dyna voltage was in the permissible range. Data is representative of one out of two independent acquisitions. The insets show the α-helical content as derived as described in Methods (see Eq. 14). MRE: molar residue ellipticity.

We then recorded the far-UV CD spectra in the presence of zinc (Fig. 10). The addition of 2.5 mM ZnSO_4_ does not trigger a gain in α-helical structure, but rather, leads to a decrease in the amplitude of the negative peak at 200 nm, consistent with a decrease in disorder content. By combining 10% TFE and 2 mM ZnSO_4_, a much more pronounced gain of structure is observed (Fig. 10), resulting in an increase in α-helical content, as judged from the appearance of the characteristic double minima at 208 and 222 nm (Fig. 10). While the addition of Zn^2+^ and 10% TFE does not promote an additional gain in α-helicity in HvASR1 (Fig. 10A), the reverse is observed for TtASR1, where the effects appear cumulative (Fig. 10B). These differences in folding propensities might reflect subtle functional differences between the two proteins. In agreement with a zinc-induced gain of structure, zinc binding also results in a decreased susceptibility of both ASR1 proteins to trypsin digestion (data not shown), as already reported for tomato ASR1^11,59^.

To gain further insights into the precise character of this structural transition, and to precisely identify the protein region where this transition takes place, we performed Hydrogen/Deuterium eXchange-Mass Spectrometry (HDX-MS) analysis.

The unlabeled ASR1 proteins were first digested with both *A*. *saitoi* protease Type XIII and pepsin to obtain peptides for monitoring local-level effects. Sequence coverage maps of all peptides of TtASR1 and HvASR1 selected for HDX-MS analysis are displayed in Supplementary Fig. S8. Excellent sequence coverage was achieved in both cases. Digestion of TtASR1 gave rise to 33 unique peptides identified from their accurate masses and product ion spectra. A total of 20 peptides were brought forward for HDX data analysis, corresponding to a sequence coverage of 92.2% (Supplementary Fig. S8). Similarly, for HvASR1, from a total of 29 identified peptides, 21 were selected for HDX-MS analysis, covering 93.7% of the total protein sequence (Supplementary Fig. S8). Amide hydrogen exchange rates within a protein are determined by both the solvent accessibility and structure of the protein. Those amide hydrogens which are fully solvent exposed and not participating in structural elements exchange rapidly, whilst those maintaining secondary structural elements exchange more slowly, due to hydrogen bonding^61^. Accordingly, amide hydrogens can be used as a structural probe to discriminate IDRs from those regions with inherent structural features^62^.

**Figure 11:**
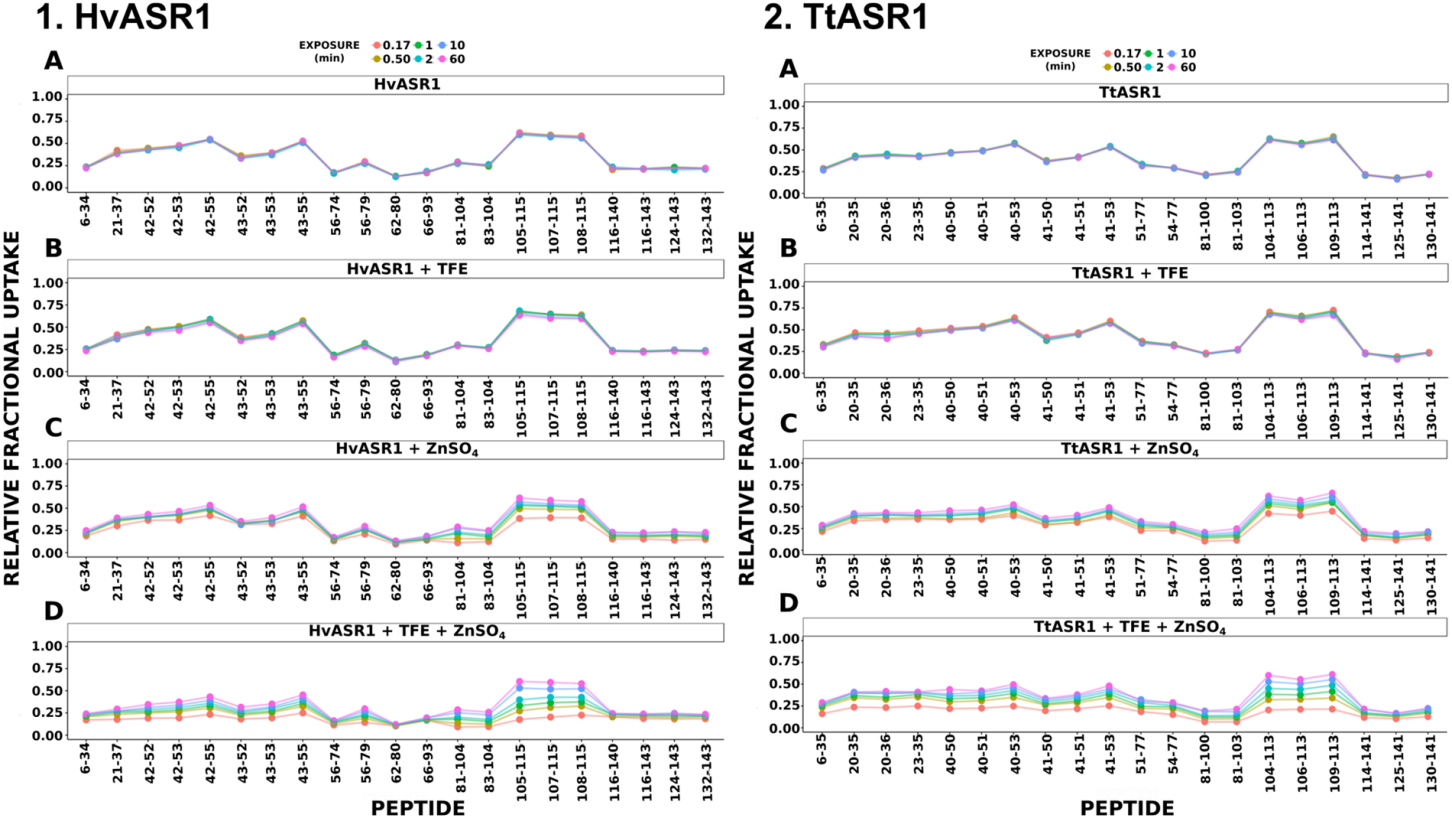
HDX-MS analysis of HvASR1 and TtASR1. The relative fractional uptake was plotted as a function of peptide position for the full-length ASR1 protein, where each color and dot represents a specific time point. The HDX-MS activity for the native proteins is displayed in panel A, in the presence of 10% TFE in Panel B, in the presence of 2 mM ZnSO_4_ in Panel C, and in the presence of both 10% TFE and 2 mM in Panel D. Both ASR1 proteins are intrinsically disordered in their native state. Structure formation occurs throughout the proteins in the presence of zinc and TFE, the most dynamic and accessible region of which being located between residues 81 to 115 for HvASR1 and 104 to 113 for TtASR1.

The HDX-MS behavior of HvASR1 was comparable to that of TtASR1, with each protein giving equivalent levels of deuterium uptake in each condition sampled (Fig. 11). In the first instance, HvASR1 and TtASR1 were found to be devoid of dynamic HDX-MS activity in the absence of either TFE or ZnSO_4_ in the timescale of the experiment (Panels A, Fig. 11). Both proteins reached their maximum exchange after 10 sec of labeling, indicating that HvASR1 and TtASR1 are predominantly unfolded in their native state. For both proteins, the presence of 10% TFE resulted in no observable dynamic HDX behavior at the equilibrium (Panels B, Fig. 11). The addition of 2 mM ZnSO_4_ induced a weak dynamic HDX-MS behavior, indicative of structure formation, throughout the entire polypeptide chain of both proteins (Panels C, Fig. 11). Notably, the addition of both 10% TFE and 2 mM ZnSO_4_ in a combined fashion resulted in an more pronounced change in magnitude of HDX dynamics (Panels D, Fig. 11). Moreover, the region spanning residues 105–115 in HvASR1, and that spanning residues 104–113 in TtASR1, are the most dynamic elements, (Panels D, Fig. 11). Furthermore, this region is also the most accessible in terms of solvent exposure.

To summarize, both TtASR1 and HvASR1 proteins behave similarly by HDX-MS. In the absence of additives, both proteins are essentially unstructured. Each protein contains a segment that is more dynamic and accessible upon zinc and TFE binding, compared to the majority of flanking sequences.

## Discussion

In the course of evolution, plants have evolved various mechanisms allowing them to adapt to abiotic stresses such as drought, low temperature, and high salinity. ID likely plays a crucial role in this ability to adapt to a varying environment. Indeed, the lack of a rigid 3D protein structure confers both binding promiscuity (ability to interact with multiple partners) and binding plasticity (ability to undergo binding-induced folding to accommodate diverse binding sites for different partners). These properties play an important role in cellular processes by conferring functional advantages in stress response, signaling and regulation^25^. In line with this, analysis of the proteome of *A*. *thaliana* indicated that approximately 23% of its proteins are mostly disordered^24^, with IDPs being over-represented in functional categories such as signaling, development, cell cycle regulation and stress response^8^. Furthermore, plant proteins exhibit significantly higher degrees of ID compared to human proteins^8^. In spite of this compelling computational evidence, experimental knowledge of plant IDPs is lacking, with only few being well characterized^24^. Dehydrins (e.g. ERD10 and ERD14) and proteins of the GRAS family are among the few plant IDPs that have been experimentally described^63,64^. Dehydrins are proteins involved in global cell protection during the highly compact dry state characteristic of plant seeds, while proteins of the GRAS family play an important role in plant development and signal transduction cascades^64^. Among the most intensively studied plant IDPs are LEA proteins^8^. The induction of expression under water deficit and/or osmotic stress are features shared by LEA and ASR proteins^10^. In addition, HvASR1 and TtASR1 can be considered as hydrophilins, as they have a high content in Gly (10%) and a high hydrophilicity (54%). LEA proteins comprise the largest group of hydrophilins. Accordingly, various studies have proposed to classify ASR proteins within this superfamily^13,15,16^, but this remains debatable^65^.

In an effort to provide additional insights into the ASR family, we have focused on the characterization of two ASR1 proteins encoded by genes that we have recently isolated. In the current study, we not only show that HvASR1 and TtASR1 are consistently predicted to be disordered by several predictors, but we also provide experimental evidence for their disordered state. In particular, we show that (i) they have an aberrant electrophoretic migration, which is a hallmark of protein disorder, (ii) they have Stokes radii, inferred from SEC that are close to the values expected for PMG conformations, (iii) they lack highly populated secondary and tertiary structure as inferred from both far- and near-CD, (iv) they lack hydrophobic cavities, as judged from DSF, (v) they are hypersensitive to proteolysis and (vi) they possess hydrodynamic parameters and SAXS scattering profiles typical of IDPs, and finally, (vii) they have no HDX-MS dynamic behavior.

Our conformational studies show that HvASR1 and TtASR1 have very similar hydrodynamic radii and radii of gyration. In agreement with this, and also with the high similarity in their charges, they have the same electrophoretic behavior in native PAGE (data not shown). The spectroscopic and hydrodynamic parameters of HvASR1 and TtASR1 indicate that, in spite of their overall extended nature, these proteins are not fully unfolded, but rather, conserve some residual compactness due to the presence of transiently populated secondary and/or tertiary structure. In addition, the R_g_ distribution of the selected sub-ensembles of HvASR1 and TtASR1 suggests the existence of two different populations characterized by a different average size. The two ASR1 proteins thus sample in solution two different subpopulations differing in their extent of compactness.

Previous studies have shown that desiccation triggers a conformational transition from an unfolded to an α-helical conformation in tomato ASR1 and MpASR proteins^10,11^. Here, using CD studies in the presence of glycerol, we show that this property is also conserved in HvASR1 and TtASR1. This latter feature constitutes an additional point of commonality with LEA proteins^52,53^. Addition of Zn^2+^ ions triggers a conformational transition from an unfolded to an α-helical conformation in tomato ASR1 and MpASR proteins^10,11^. By contrast, in the case of soybean ASR protein (GmASR), Zn^2+^ was shown to induce (reversible) aggregation. The authors speculated that the expression of GmASR might be up regulated to buffer the concentration of Zn^2+^, thus alleviating metal toxicity under stressed conditions^66^. In the case of tomato ASR1, addition of Zn^2+^ ions not only triggers a gain of structure, but also induces homodimerization^11,59^. Note that we could not assess possible Zn-induced homodimerization of ASR1 proteins, as the addition of ZnSO_4_ in SEC systematically led to the precipitation of the protein onto the column.

While it is plausible that the Zn-induced structural transition observed in HvASR1 and TtASR1 plays a role in DNA-binding, as observed for tomato ASR1^59^, it is also tempting to speculate that it can also serve to protect membranes against drought or freezing, by analogy with other LEA proteins that were shown to fold upon dehydration and to bind to membranes in their folded state^67^. HDX-MS studies allowed us to precisely identify a region common to both proteins which is more dynamic and solvent exposed after addition of Zn^2+^ than the rest of the protein (see underlined fragment in Fig. 8). In the case of HvASR1, this region extends to include residues 81–104. Notably, this region contains an amino acid stretch (PEHAHKHK) that is also conserved in tomato ASR1, and that was previously shown to bind to zinc^59^.

According to CD analyses, TtASR1 and HvASR1 undergo α-helical folding in the presence of TFE. This likely reflects their inherent propensity to undergo conformational change as part of their physiological function, as for instance, during ligand binding. Even if TFE is known to stabilize α-helices more than β-strands, it is worth emphasizing that gain of α-helicity in the presence of TFE is not a general rule and hence truly reflects the inherent structural propensities of the protein under study. For instance, (i) the acidic activator domain of GCN4 forms little or no α-helix in TFE concentrations as high as 30%, and folds mostly as β-sheets in 50% TFE^68^, and (ii) the intrinsically disordered dehydrin Rab18 has an α-helical content as low as 2% in the presence of 90% TFE^69^. To further support the argument that TFE does not promote non-native folding, the intrinsically disordered N_TAIL_ domains undergo α-helical folding in the presence of 20% TFE exclusively within a region known to undergo partner-induced α-helical folding^70,71^. All of these considerations argue that TFE has the ability to increase the structural propensity of IDPs.

Many IDPs undergo (partial) folding upon binding to their partner(s)/ligand(s), with short, transiently structured regions, referred to as MoREs, being primarily involved in these events^32^. We show that HvASR1 and TtASR1 possess short regions locally enriched in hydrophobic clusters that correspond to predicted MoREs. The limited proteolysis experiments carried out in the presence of TFE allowed the identification of a resistant fragment in both ASR1 proteins. Interestingly, this fragment contains a predicted α-helix and a predicted MoRE, and also overlaps with the region that becomes more structured upon addition of Zn^2+^. It therefore corresponds to a *bona fide* MoRE undergoing folding coupled to zinc binding.

Given the increasing interest that is being paid to IDPs, and the paucity of studies pertaining the structural characterization of plant IDPs in general, and of ASR proteins in particular, this work represents an important contribution that sheds light on fundamental aspects of plant IDPs. Unraveling the complex biology of multifunctional ASR proteins is challenging. The present study, by providing the first experimental characterization of two ASR1 proteins and by unveiling their disordered nature, paves the way towards future studies that will reveal how ASR functional regulations and involvement in stress response is achieved *via* their flexibility. It also sets the basis for achieving atomic-resolution conformational ensemble description of these two plant IDPs. Finally, taking into account previous reports that established a relationship between structural disorder and protein interactivity, the present results suggest that HvASR1 and TtASR1 proteins are likely to be involved in manifold protein-protein and/or protein-DNA interactions.

## Methods

### Disorder predictions and in silico analysis of amino acid composition

Sequence accession numbers are KX660743 for HvASR1 and KX660744 for TtASR1. Disordered regions within HvASR1 and TtASR1 were identified using the MeDor (http://www.vazymolo.org/MeDor/)^72^, and the Genesilico MetaDisorder (http://iimcb.genesilico.pl/metadisorder/metadisorder.html)^73^ metaservers for the prediction of disorder. These metaservers collect disorder and secondary structure predictions from servers available on the web. Beyond canonical predictors, MeDor also incorporates HCA^74^. DorA (Disorder Analyser) is a predictor that identifies regions of disorder based on the combined use of a disorder scoring matrix and HCA. It works by first detecting regions that exhibit an amino acid composition bias towards disorder-promoting residues and then by filtering them to eliminate segments rich in hydrophobic clusters.

Low complexity regions were identified using SEG (http://mendel.imp.ac.at/METHODS/seg.server.html)^75^, using trigger window length [W] = 12, trigger complexity [K(1)] = 2.2 and extension complexity [K(2)] = 2.5.

Disordered binding regions, i.e. regions with a propensity to undergo folding upon binding to a partner, were identified using ANCHOR (http://anchor.enzim.hu/)^76^ and MoRFpred (http://biomine-ws.ece.ualberta.ca/MoRFpred/index.html)^77^.

HvASR1 and TtASR1 were also submitted to charge/hydropathy analysis, a binary predictor allowing globular proteins to be distinguished from unstructured ones based on the ratio of their net charge (R) *versus* their hydropathy (H)^18^. A protein is predicted as disordered if H < [(R + 1.151)/2.785]. The RH-plot was generated by choosing this option on the main page of the PONDR server (http://www.pondr.com/cgi-bin/PONDR/pondr.cgi). Analysis of sequence attributes, as defined in^38^, were carried out using the CIDER server (http://pappulab.wustl.edu/CIDER/). The letter was also used to generate the phase diagram plots.

Deviations in amino acid composition of HvASR1 and TtASR1 were analyzed and computed as already described^78^ using the average amino acid frequencies of the SWISS-PROT database (as obtained from http://us.expasy.org/sprot) as the reference value. The average amino acid frequencies of the SWISS-PROT database roughly corresponds to the mean composition of proteins in nature. If the average composition of an amino acid X in SWISS-PROT proteins is CSP_X_, and CP_X_ is the composition of X within a protein P, deviation from the composition of X of SWISS-PROT proteins was defined for P as (CP_X_-CSP_X_)/CSP_X_.

### Proteins expression and purification

The full-length TtASR1 and HvASR1 cDNAs, encoding a protein of 136 and 138 residues respectively, were cloned in the pGEX4T-1 expression vector into the EcoRI site using standard restriction and ligation techniques. This vector allows the bacterial expression of the protein of interest as a fusion protein appended to a cleavable glutathione S transferase (GST) tag. Removal of the GST tag by thrombin results in a protein with an N-terminal, vector-encoded GSPEF amino acid extension. The sequence of the coding region was checked by sequencing performed in center of biothechnology of sfax (CBS) analysis service using the automated sequencer (ABI PRISM 3100 PRE) and found to conform to expectations.

The *E*. *coli* strain BL21 was used for the expression of the recombinant proteins. Cultures were grown overnight to saturation in LB medium containing 100 µg mL^−1^ ampicillin. An aliquot of the overnight culture was diluted 1/50 into 1 L of LB medium and grown at 37 °C. When the optical density at 600 nm (OD_600_) reached 0.5–0.8, isopropyl β-D-thiogalactopyranoside (IPTG) was added to a final concentration of 0.5 mM, and the cells were grown at 37 °C for 4 additional hours. The induced cells were harvested, washed and collected by centrifugation (4000 rpm, 20 min). The resulting pellets were frozen at −20 °C.

Each bacterial pellet was resuspended in 25 mL of buffer A (50 mM sodium phosphate pH 7, 300 mM NaCl) supplemented with 0.1 mg mL^−1^ lysozyme, 10 μg mL^−1^ DNAse I, MgSO_4_ 20 mM and half a tablet of EDTA-free protease inhibitor cocktail (Sigma). After 20 min of incubation with gentle agitation, the cells were disrupted by sonication (using a 750 W sonicator and 3 cycles of 30 s each at 45% power output). The lysates were clarified by centrifugation at 14000 rpm for 30 min at 4 °C. The clarified supernatant was incubated with 1 mL of glutathione Sepharose 4B resin (GE, Healthcare), previously equilibrated with buffer A, for 1 h with gentle shaking at 4 °C. Then, the resin was washed three times with buffer A. To remove the GST-tag, 1 mL of Phosphate Buffered Saline (PBS) pH 7.3 containing 5 U of bovine thrombin (Calbiochem) was added to the resin. After a 16 h incubation at 22 °C, the non-retained fraction was recovered by pelleting the resin. To ensure optimal recovery of the protein of interest from the resin, the latter was washed with 2 mL of PBS, thus yielding a final volume of 3 mL. The presence of the protein was checked by SDS-PAGE. Each protein was further purified by SEC. To this end, the sample (typically 6 mL at 2.5 mg/mL) was loaded onto a Superdex 75 16/60 column (GE, Healthcare) using a fast protein liquid chromatography (FPLC) Äkta system (GE, Healthcare) and eluted in PBS pH 7.3.

Protein concentrations were calculated using the theoretical absorption coefficients at 280 nm as obtained using the program ProtParam at the EXPASY server. For both proteins, typical purification yields were of 0.5 mg of purified protein *per* liter of bacterial culture.

### Size Exclusion Chromatography and calculation of hydrodynamic radii

The hydrodynamic radii (Stokes radii, R_S_) of the HvASR1 and TtASR1 proteins were estimated by analytical SEC. Typically 2.6 mg mL^−1^ of purified protein was injected. The SEC buffer was PBS pH 7.3.

The Stokes radii of proteins eluted from the SEC column were deduced from a calibration curve obtained using globular proteins of known molecular mass (MM, in Daltons) and whose R_S_ (in Å) was calculated according to^79^:

1 $${\rm{log}}({{R}_{S}}^{{\rm{Obs}}})=0.369* ({\rm{log}}\,{\rm{MM}})-0.254$$

The R_S_ (in Å) of a natively folded (Rs^NF^), fully unfolded state in urea (R_S_^U^) and natively unfolded premolten globule (PMG) (R_S_^PMG^) protein with a molecular mass (MM) (in Daltons) were calculated according to^20^:

2 $${\rm{log}}({{R}_{S}}^{{\rm{NF}}})=0.357\,* ({\rm{log}}\,{\rm{MM}})-0.204$$

3 $${\rm{log}}({{R}_{S}}^{{\rm{U}}})=0.521\,* \,{\rm{log}}({\rm{MM}})-0.649$$

4 $${\rm{log}}({{R}_{S}}^{{\rm{PMG}}})=0.392\,* ({\rm{log}}\,{\rm{MM}})-0.210$$

5 $${\rm{log}}({{R}_{S}}^{{\rm{MG}}})=0.334\,* ({\rm{log}}\,{\rm{MM}})-0.053$$

The R_S_ (in Å) of a natively folded dimeric form (Rs^DimNF^), was calculated as:

6 $${\rm{log}}({{R}_{S}}^{{\rm{Dim}}{\rm{NF}}})=0.357\,* ({\rm{log}}\,{\rm{MM}}* 2)-0.204$$

The R_S_ of an IDP with N residues was also calculated according to^80^ using the simple power-law model:

7 $${{{\rm{R}}}_{{\rm{S}}}}^{{\rm{IDP}}}={{\rm{R}}}_{{\rm{0}}}{{\rm{N}}}^{{\rm{\nu }}}$$

where R_0 = _2.49 and ν = 0.509.The compaction index (CI) is expressed as according to^81^:

8 $${\rm{CI}}=(R{s}^{{\rm{U}}}-R{s}^{{\rm{obs}}})/(R{s}^{{\rm{U}}}-R{s}^{{\rm{NF}}})$$

This parameter, which allows comparison between proteins of different lengths, can vary between 0 and 1, with 0 indicating minimal compaction and 1 maximal compaction.

### Native ESI-MS analysis

We carried out native electrospray ionization mass spectrometry (ESI-MS) studies to decipher the macromolecular composition of TtARS1 and HvASR1, *i*.*e*. to assess whether they are monomers or dimers. As a control, we first measured ADH to confirm that the MS parameters used were suitable to maintain non-covalent complexes. Prior to analysis, all proteins were buffer-exchanged into 250 mM ammonium acetate, pH 8.0 using Micro Bio-Spin^TM^ 6 Columns (Bio-Rad). All proteins were infused at a concentration of 15 μM. Native ESI-MS was performed on a Synapt G2-Si mass spectrometer (Waters). The capillary voltage was set to 1.8–2.1 kV, the source temperature was 30 °C, the sampling cone was set to 150 V, the source offset to 150 V, and the trap gas flow to 4 mL/min. The latter was adjusted to 5 mL/min for ADH.

### Differential scanning fluorimetry (DSF)

DSF monitors thermal unfolding of proteins in the presence of a fluorescent dye^82^. A solution of SYPRO Orange (5000× stock solution) was diluted in water to yield a 7× working solution. This experiment was conducted using a PCR instrument (Biorad) and 96-well plates containing 25 µL of mixture *per* well. Each well contained 21.5 µL of protein solution (HvASR1 and TtASR1 at 1 mg mL^−1^) and 3.5 µL of SYPRO Orange working solution. Fluorescent signals were acquired with excitation and emission wavelengths at 485 nm and 625 nm, respectively. Temperature scans were performed from 20 °C to 90 °C.

### SAXS measurements and calculation of the radius of gyration

All SAXS measurements were carried out at the European Synchrotron Radiation Facility (ESRF) on beamline BM29 at a working energy of 12.5 KeV. The sample-to-detector distance of the X-rays was 2.847 m, leading to scattering vectors *q* ranging from 0.028 to 4.525 nm^−1^. The scattering vector is defined as *q* = 4π/λ sin*θ*, where 2θ is the scattering angle. The exposure time was optimized to reduce radiation damage.

SAXS data were collected at 20 °C using purified protein samples (30 μL each). Protein concentrations were as follows: 1.0 and 1.5 g/L for HvASR1 and 1.0 and 1.5, 2.0 and 3.0 g/L for TtASR1. Both proteins were in PBS pH 7.3 buffer containing 5 mM DTT.

Samples were loaded in a fully automated sample charger. Ten exposures of 10 s each were made for each protein concentration and data were combined to give the average scattering curve for each measurement. Any data points affected by aggregation, possibly induced by radiation damages were excluded. The profiles obtained in the range 1.0–1.5 g/L for HvASR1, and 1.0–3.0 g/L for TtASR1 had the same shape and were flat at low q values indicating the absence of significant aggregation. Then, we used the higher concentration (1.5 g/L for HvASR1 and 3.0 g/L for TtASR1) to obtain maximal information at high resolution.

The data were analyzed using the ATSAS program package^83^. Data reductions were performed using the established procedure available at BM29, and buffer background runs were subtracted from sample runs. The Rg and forward intensity at zero angle I(0) were determined with the program PRIMUS^84^ according to the Guinier approximation at low q values, in a q.Rg range up to 1.3:

9 $$Ln[I(Q)]=Ln[{I}_{0}]-\frac{{Q}^{2}{R}_{g}^{2}}{3}$$

The forward scattering intensities were calibrated using bovine serum albumin as reference. The Rg and pair distance distribution function, P(r), were calculated with the program GNOM^85^. The maximum dimension (D_max_) value was adjusted such that the Rg value obtained from GNOM agreed with that obtained from the Guinier analysis.

A pool of random-coil conformers was generated using Flexible-Meccano^86^. An optimized sub-ensemble of conformations that agrees with the experimental scattering curve was selected from the large conformational ensemble using GAJOE^87^. The maximum size of the final optimized ensemble was set to 50.

The theoretical value of Rg (in Å) expected for an IDP was calculated using Flory’s equation according to^46^:

10 $${\rm{Rg}}={{\rm{R}}}_{{\rm{0}}}{{\rm{N}}}^{{\rm{\nu }}}$$

where N is the number of amino acid residues, R_0_ is 2.54 ± 0.01 and ν is 0.522 ± 0.01.

The theoretical value of Rg (in Å) expected for a globular protein was calculated according to^45^:

11 $${{{\rm{R}}}_{{\rm{g}}}}^{{\rm{NF}}}=\sqrt{(3/5)4.75\,{{\rm{N}}}^{0.29}}$$

The theoretical value of Rg (in Å) expected for a fully unfolded form was calculated according to^45^:

12 $${\rm{Log}}({{{\rm{R}}}_{{\rm{g}}}}^{{\rm{U}}})=0.58\,{\rm{Log}}({\rm{N}})+0.80$$

The theoretical radius of gyration (R_g_, in Å) expected for a globular protein with a hydrodynamic radius R_S_ was calculated according to^45^:

13 $${{{\rm{R}}}_{{\rm{g}}}}^{{\rm{NF}}}={(3/5)}^{1/2}{{\rm{R}}}_{{\rm{S}}}$$

HvASR1 and TtASR1 consist of 143 and 141 residues, respectively, including vector-encoded residues. Using an average volume of 134 Å^3^ *per* residue for proteins, the radius of a sphere with volume V = 4/3 πR_S_^3^ would be 16.6 Å in the case of HvASR1, and 16.5 Å in the case of TtASR1. According to Eq. 13, the corresponding R_g_ would be 12.8 Å for both ASR1 proteins.

### Circular dichroism (CD) measurements

CD spectra of HvASR1 and TtASR1 were measured using a Jasco 810 dichrograph, flushed with N_2_ and equipped with a Peltier thermoregulation system. One-mm or 1-cm thick quartz cuvettes were used for far- and near-UV CD measurements, respectively. Proteins concentrations were 0.1 mg mL^−1^ and 1 mg mL^−1^ for far- and near-UV CD studies, respectively. Far-UV CD spectra were measured between 190 and 260 nm, while near-UV CD spectra were recorded between 250 and 350 nm. Unless differently specified, CD spectra were recorded in 10 mM sodium phosphate pH 7 at 20 °C. The scanning speed was 20 nm/min, with data pitch of 0.2 nm. Each spectrum is the average of three acquisitions. The spectrum of buffer was subtracted from the protein spectrum. Spectra were smoothed using the “means-movement” smoothing procedure implemented in the Spectra Manager package.

Far-UV CD spectra were also recorded in the presence of increasing concentrations (from 20 to 50%) of TFE. Mean molar ellipticity values per residue (MRE) were calculated as (Θ) = 3300MΔA/(lcn), where l is the path length in cm, n is the number of residues, M is the molecular mass in Daltons and c is the concentration of the protein in mg mL^−1^. Numbers of amino acid residues are 141 for TtASR1 and 143 for HvASR1. Molecular masses are 15,645 Da for TtASR1 and 15,922 Da for HvASR1.

In order to study protein unfolding, measurements at a fixed wavelength of 230 nm were performed in the temperature range of 20 °C–80 °C, with data pitch 1 °C and a temperature slope of 1 °C/min and protein concentrations of 0.1 mg mL^−1^.

The DICHROWEB website (http://dichroweb.cryst.bbk.ac.uk/html/home.shtml), which was supported by grants to the BBSRC Centre for Protein and Membrane Structure and Dynamics (CPMSD)^88^, was used to analyze the experimental data in the 190–260 nm range. The content in the various types of secondary structure was estimated using the CDSSTR deconvolution method with the reference protein set 7^89^.

The α-helical content was estimated as follows:

For the estimation of the percentage of residues adopting an α-helical conformation in the presence of various additives, such as glycerol, TFE, sucrose and ZnSO_4_, we analyzed the value of molar ellipticity at 220 nm observed under each condition and divided it by the value expected for a protein whose all residues adopt an α-helical conformation (100% α-helix). The latter was calculated according to the following empirical relationship^90^:1where n is the number of residues.

### Limited proteolysis by thermolysin

Limited proteolysis was used to identify possible folded fragments within the HvASR1 and TtASR1 proteins. The proteins (at 1 mg mL^−1^) were incubated with thermolysin in 20 mM Tris/HCl pH 7.8 at 25 °C supplemented with 15% TFE. A thermolysin stock solution at 800 µg mL^−1^ was used in this experiment. Protease to protein substrate ratios were 1:100 (w/w). The extent of proteolysis was evaluated by SDS–PAGE analysis of 20 µL aliquots removed from the reaction mixture over a time course (0, 1, 3, 6 and 24 hours), added to 5 µL of 5x loading sample buffer and boiled for 5 min to inactivate the protease. Proteins incubated with only thermolysin without TFE were used as controls.

### Mass spectrometry (MALDI-TOF)

Mass analysis of the purified ASR1 proteins was performed using a MALDI-TOF-TOF Bruker Ultraflex III spectrometer (Bruker Daltonics, Wissembourg, France) controlled by the Flexcontrol 3.0 package (Build 51). This instrument was used at a maximum accelerating potential of 25 kV and was operated in the linear mode with *m/z* range from 600 to 3500. Samples (1 µL containing 15 pmol) were mixed with an equal volume of α-Cyano-4-hydroxycinnamic acid matrix solution, spotted on the target, then dried at room temperature for 10 min.

Mass spectral analysis of the protein fragments, as obtained upon thermolysin limited digestion of the purified HvASR1 and TtASR1 proteins, was performed as follows. After SDS-PAGE separation, the bands of interest were excised from the gel. The bands were then digested with trypsin (0.25 μg trypsin per μg of protein substrate). For each protein band, mass analyses were performed on a MALDI-TOF-TOF Bruker Ultraflex III spectrometer as described above, except that the instrument was operated in reflector mode. The mass standards were either autolytic tryptic peptides used as internal standards or peptide standards (Bruker Daltonics). Following MS analysis, MS/MS analyses were performed on the most intense peaks to identify the amino acid sequence of the protein band.

### Binding of HvASR1 and TtASR1 proteins to Zn and Ni

The ability of both HvASR1 and TtASR1 proteins to bind to zinc and nickel ions was assessed by incubating the purified proteins (25 μL at 1 mg/mL in PBS) with 60 μL of a sepharose fast-flow resin previously loaded with either ZnSO_4_ or NiSO_4_ (100 mM each) and equilibrated in PBS. After a 3 hour incubation with gentle agitation at 4 °C, the resin was washed (4 times with 1 mL of PBS) and analyzed by SDS-PAGE by loading 12 μL.

### Fluorescence spectroscopy

Fluorescence spectra of the Tyr residues in both ASR1 proteins were recorded by using a Cary Eclipse (Varian) equipped with a front face fluorescence accessory at 20 °C, with 5 nm excitation and 5 nm emission bandwidths. The excitation wavelength was 280 nm, and the emission spectra were recorded between 280 and 400 nm. Spectra were recorded using a 1-ml quartz fluorescence cuvette containing 1 mg/mL of protein in PBS. ZnSO_4_ was directly added to the protein solution to reach the desired concentration.

### HDX-MS analyses

For each of the two ASR1 proteins, we monitored various conditions: A) ASR1 protein alone, B) ASR1 in the presence of 10% TFE, C) ASR1 in the presence of 2 mM ZnSO_4_ and D) ASR1 in the presence of both 10% TFE and 2 mM ZnSO_4._ Prior to addition of the deuterated buffer, all solutions were equilibrated for 1 h at room temperature. Continuous labeling was performed at 20 °C for *t* = 0.16, 0.5, 1, 2, 10, and 60 min. The labeling reaction for each experimental condition was performed using a deuterium solution supplemented with either TFE or ZnSO_4_, as above. The final deuterium concentration in each experiment was 72%. Aliquots of 10 pmol of protein were withdrawn at each experimental time point and quenched upon mixing of the deuterated sample with ice-cold 0.5% formic acid solution, achieving a final pH of 2.5. Quenched samples were immediately snap-frozen in liquid nitrogen and stored at −80 °C for approximately 24 h. Undeuterated controls were prepared using an identical procedure. Triplicate analyses were performed for each time point and condition for all HDX-MS analyses.

Prior to MS analysis, samples were rapidly thawed on ice. To minimize back exchange, the LC solvent line, injection valve, and sample loop were maintained at 0 °C with the aid of a cooled HDX Manager (Waters Corporation, Milford, MA). For peptide analysis, samples were initially digested with protease Type XIII from *Aspergillus saitoi* for 5 min on ice, followed by sequential digestion using an in-house prepared cartridge of immobilized pepsin beads (Thermo Scientific, Rockford, IL), for 2 min at 100 µL/min and 20 °C. Peptic peptides were rapidly desalted and concentrated using a Vanguard C4 pre-column (1.7 µm, 2.1 × 5 mm; Waters), and separated using an ACQUITY UPLC™ BEH C18 column (1.7 µm, 1 × 100 mm). ASR1 peptides were separated over a 10 min gradient of 5–40% ACN at 40 µL/min and at 0 °C. The LC flow was directed to a Synapt™ G2-Si HDMS™ mass spectrometer (Waters) that was equipped with ESI and lock-mass correction using Glu-Fibrinogen peptide. Mass spectra were acquired in positive-ion mode over the *m/z* range of 50–1800 using a data-independent acquisition scheme (MS^E^) whereby exact mass information is collected at both low and high collisional energies for collisional induced dissociation. To enable local-level analysis, unlabeled TtASR1 and HvASR1 samples were digested in duplicate to build a peptide coverage map.

Peptide identification was *via* the Protein Lynx Global Server (PLGS) 3.0 (Waters). Oxidation of methionines and carbamylation of N-terminal and lysine residues were set as variable modifications. The sequence coverage map was plotted using DynamX 3.0 HDX software (Waters). D_2_O uptake at the peptide level was extracted and visualized in uptake charts, difference plots, and heat maps, also performed in DynamX. HDX-MS results were further analyzed using MEMHDX^91^.

### Data availability statement

All data herein presented are available upon request.

## Electronic supplementary material

Supplementary Information
