# Supplementary material for: Structural disorder and induced folding within two cereal, ABA stress and ripening (ASR) proteins
Source: Sci Rep. 2017 Nov 14;7:15544. doi: 10.1038/s41598-017-15299-4 (PMC5686140; doi:10.1038/s41598-017-15299-4)
Supplement: Supplementary file 1 — Supplementary Information [file 41598_2017_15299_MOESM1_ESM.pdf]

Supplementary Information

Structural disorder and induced folding within two cereal, ABA stress and ripening (ASR) proteins

Karama Hamdi[1], Edoardo Salladini[2], Darragh P. O'Brien[3], Sébastien Brier[4], Alexandre Chenal[3], Ines Yacoubi[1*] and Sonia Longhi[2*]

[1]Laboratoire de Protection et d'Amélioration des Plantes, Centre de Biotechnologie de Sfax (CBS), Tunisia
[2]Aix-Marseille Univ, CNRS, Architecture et Fonction des Macromolécules Biologiques (AFMB), UMR 7257, Marseille, France
[3]Institut Pasteur, CNRS UMR 3528, Unité de Biochimie des Interactions Macromoléculaires, Département de Biologie Structurale et Chimie, Paris, France
[4]Institut Pasteur, CNRS USR 2000, Unité de Spectrométrie de Masse Structurale et Protéomique, Paris, France

*to whom correspondence should be sent

Sonia Longhi
AFMB, UMR 7257 CNRS and Aix-Marseille University
163, avenue de Luminy, Case 932, 13288 Marseille Cedex 09, France
Tel: (33) 4 91 82 55 80; Fax: (33) 4 91 26 67 20
E-mail: Sonia.Longhi@afmb.univ-mrs.fr

Inès Yacoubi
Biotechnology and Plant Improvement Laboratory
Centre of Biotechnology of Sfax (CBS), University of Sfax
Street Sidi Mansour Km 6, P.O. Box "1177", 3018, Sfax, Tunisia
E-mail: ines.yacoubi@yahoo.com

Supplementary Text 1
DorA is a disorder predictor that identifies regions of disorder based on the combined use of a disorder scoring matrix and HCA, while FoldIndex is a derivative of the charge/hydropathy method that provides positional information [1].

Supplementary Text 2
For globular proteins, gradually increasing the temperature triggers unfolding and concomitant exposure of hydrophobic cavities in folded proteins, thereby leading to an increase in fluorescence. When the fluorescence intensity is plotted as a function of the temperature, the apparent melting temperature (Tm) of the protein can be derived from the inflection point of the resulting sigmoidal curve. By contrast, IDPs, which do not possess hydrophobic cavities being devoid of a stable 3D structure, are characterized by a fluorescence profile that is rather flat and temperature-independent.

Supplementary Text 3
A very useful method to describe the structural properties of a molecule is the Kratky plot. In particular, from the shape of this plot the conformation adopted by the molecule can be inferred. The Kratky plot of a globular protein has a bell shape with a clear maximum. For completely unfolded proteins or for a PMG, no such maximum can be observed, and the curve displays a plateau [2].

Supplementary Text 4
TFE stabilizes secondary structures by strengthening peptide hydrogen bonds in TFE/H_2O mixtures and through favorable interactions of hydrophobic amino acid side chains with TFE.

Supplementary Text 5
Near-UV CD is a well-suited technique to monitor small conformational changes since the CD contributions of the aromatic side chains are usually very sensitive to their environment [3]. Furthermore, aromatic amino acid tends to have a characteristic wavelength profile [3].

References

1 Zeev-Ben-Mordehai, T. *et al.* The intracellular domain of the Drosophila cholinesterase-like neural adhesion protein, gliotactin, is natively unfolded. *Proteins-Structure Function and Bioinformatics* **53**, 758-767, doi:10.1002/prot.10471 (2003).
2 Bernado, P. & Svergun, D. I. Structural analysis of intrinsically disordered proteins by small-angle X-ray scattering. *Mol Biosyst* **8**, 151-167, doi:10.1039/c1mb05275f (2012).
3 Kelly, S. M., Jess, T. J. & Price, N. C. How to study proteins by circular dichroism. *Biochim Biophys Acta* **1751**, 119-139, doi:10.1016/j.bbapap.2005.06.005 (2005).

Legends of Supplementary Figures

Figure S1. Amino acid composition analysis of HvASR1 (**A**) and TtASR1 (**B**) proteins. Deviation in amino acid composition from the Swiss-PROT data base of HvASR1 and TtASR1. The relative enrichment in disorder promoting (red bars) and order-promoting (blue bars) residues is shown. Residues have been ordered on the x axis according to the TOP-IDP flexibility index as described in [36]. The insets show the amino acid composition of the proteins.

Figure S2. Charge-hydropathy plot of HvASR1 (**A**) and TtASR1 (**B**) proteins. The mean net charge (R) is plotted against the mean hydrophobicity (H). In the left part of the CH plot, a protein is predicted to be intrinsically disordered, whereas it is predicted to be structured if it falls in the right part of it (see Materials and Methods).

Figure S3. Native ESI-MS spectrum of ADH (15 μM) in 250 mM ammonium acetate, pH 8 (Synapt G2-Si mass spectrometer, Waters) showing that the tetrameric state of ADH is preserved under the MS conditions herein used.

Figure S4. Differential scanning fluorimetry (DSF) of HvASR1 and TtASR1 in the presence of Sypro Orange in the 20-95°C temperature range.

Figure S5. SAXS studies of HvASR1. (**A**) Representation of the Guinier plot for the protein at 1.5 g/L. Inset: residuals. (**B**) Pair distance distribution, P(r), function of the data for the 1.5 g/L concentration. (**C**) Kratky plot of the SAXS data obtained at 1.5 g/L. (**D**) Log-log representation of HvASR1 and BSA SAXS patterns. (**E**) Experimental scattering curve of the protein at 1.5 g/L (red) and GAJOE fit (black). (**F**) Distribution of Rg of the ensemble of randomly generated conformers by Flexible-Meccano without constraints (red) and of the sub-ensemble of selected conformers using GAJOE (black).

Figure S6. Far-UV CD spectra of HvASR1 (**A**) and TtASR1 (**B**) in the absence or presence of 30% sucrose at 20°C. Proteins were at 0.1 mg/mL in 10 mM sodium phosphate pH 7. Data are shown up to 197 nm, point beyond which the dyna voltage was no longer in the permissible range. Data are representative of one out of two independent acquisitions. The insets show the α-helical content as derived as described in Materials and Methods (see Eq. 14). MRE: molar residue ellipticity.

Figure S7. SDS-PAGE analysis of a fast-flow sepharose resin equilibrated in PBS buffer either without (Ctrl) or with Ni^{2+} (Ni) or Zn^{2+} (Zn) ions, after incubation with the purified ASR1 proteins followed by extensive washing. Both proteins were found to bind to the resin, reflecting their ability to bind to both Ni^{2+} and Zn^{2+} ions. Input: amount of protein loaded onto the resin. MM: molecular mass markers.

Figure S8. Peptide map of HvASR1 and TtASR1. A sequence coverage map of the protein was generated using a sequential digestion strategy of both *A. saitoi* protease Type XIII and

pepsin. Each blue bar represents a single ASR1 peptide. The achieved linear sequence coverage was 93.7% for HvASR1 and of 92.2% for TtASR1.

pepsin. Each blue bar represents a single ASR1 peptide. The achieved linear sequence coverage was 93.7% for HvASR1 and of 92.2% for TtASR1.

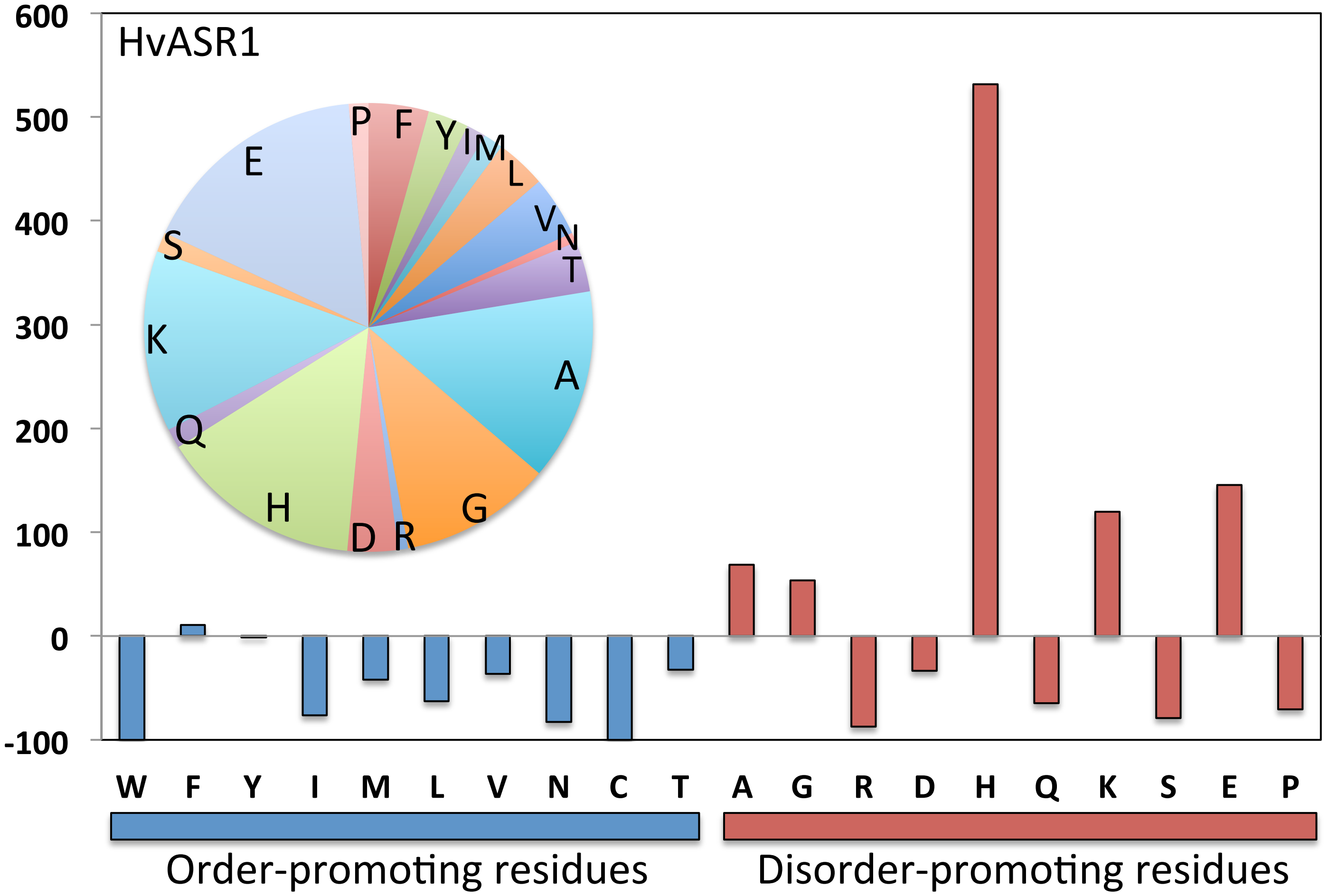
HvASR1
600
500
400
300
200
100
0
-100
P F Y I M L V N T A G R D H Q K S E
W F Y I M L V N C T A G R D H Q K S E P
Order-promoting residues
Disorder-promoting residues

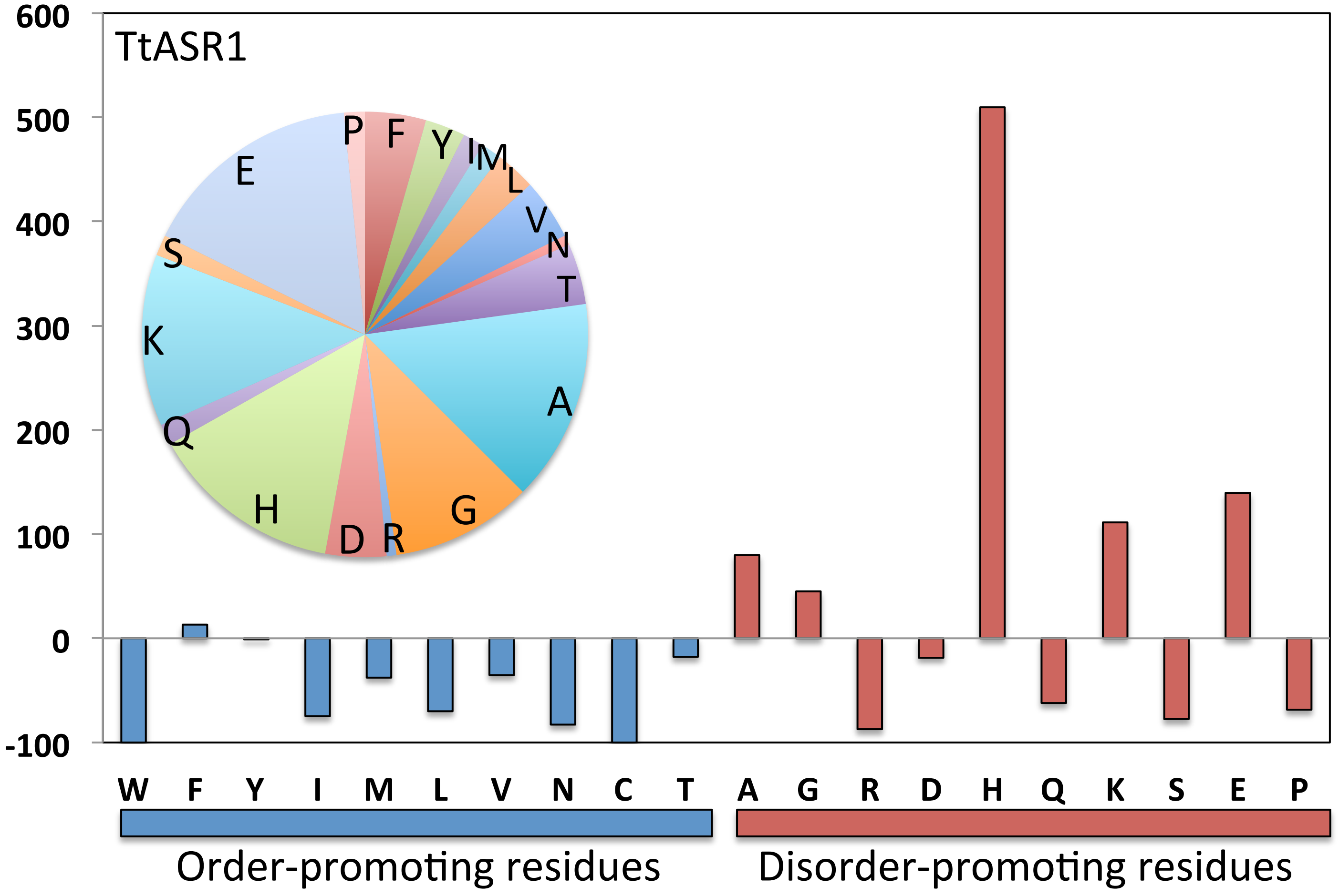
TtASR1
600
500
400
300
200
100
0
-100
P F Y I M L V N T A G R D H Q K S E
W F Y I M L V N C T A G R D H Q K S E P
Order-promoting residues
Disorder-promoting residues

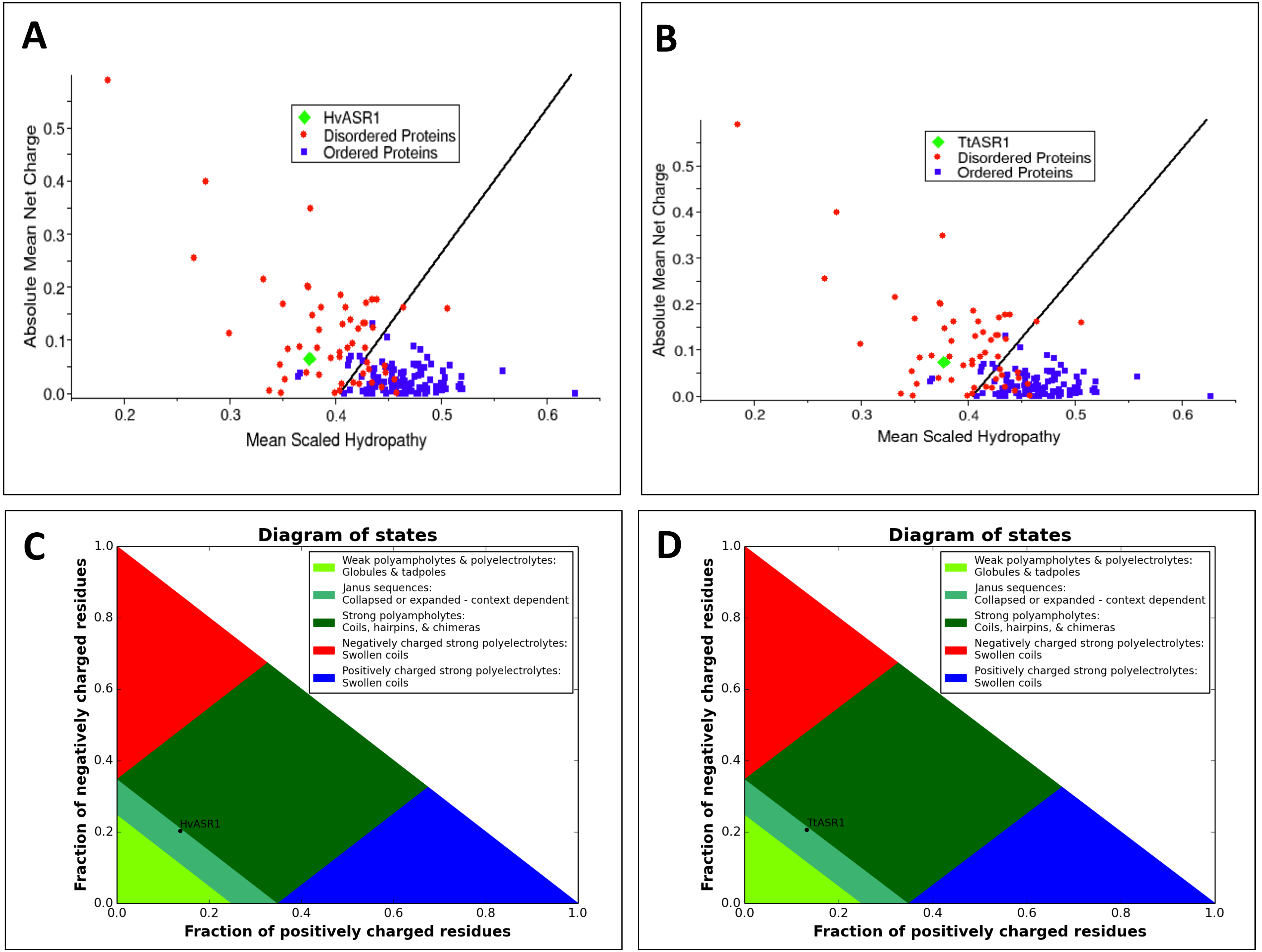
A
HvASR1
Disordered Proteins
Ordered Proteins
Absolute Mean Net Charge
Mean Scaled Hydropathy
B
TtASR1
Disordered Proteins
Ordered Proteins
Absolute Mean Net Charge
Mean Scaled Hydropathy
C
Diagram of states
Weak polyampholytes & polyelectrolytes: Globules & tadpoles
Janus sequences: Collapsed or expanded - context dependent
Strong polyampholytes: Coils, hairpins, & chimeras
Negatively charged strong polyelectrolytes: Swollen coils
Positively charged strong polyelectrolytes: Swollen coils
HvASR1
Fraction of negatively charged residues
Fraction of positively charged residues
D
Diagram of states
Weak polyampholytes & polyelectrolytes: Globules & tadpoles
Janus sequences: Collapsed or expanded - context dependent
Strong polyampholytes: Coils, hairpins, & chimeras
Negatively charged strong polyelectrolytes: Swollen coils
Positively charged strong polyelectrolytes: Swollen coils
TtASR1
Fraction of negatively charged residues
Fraction of positively charged residues

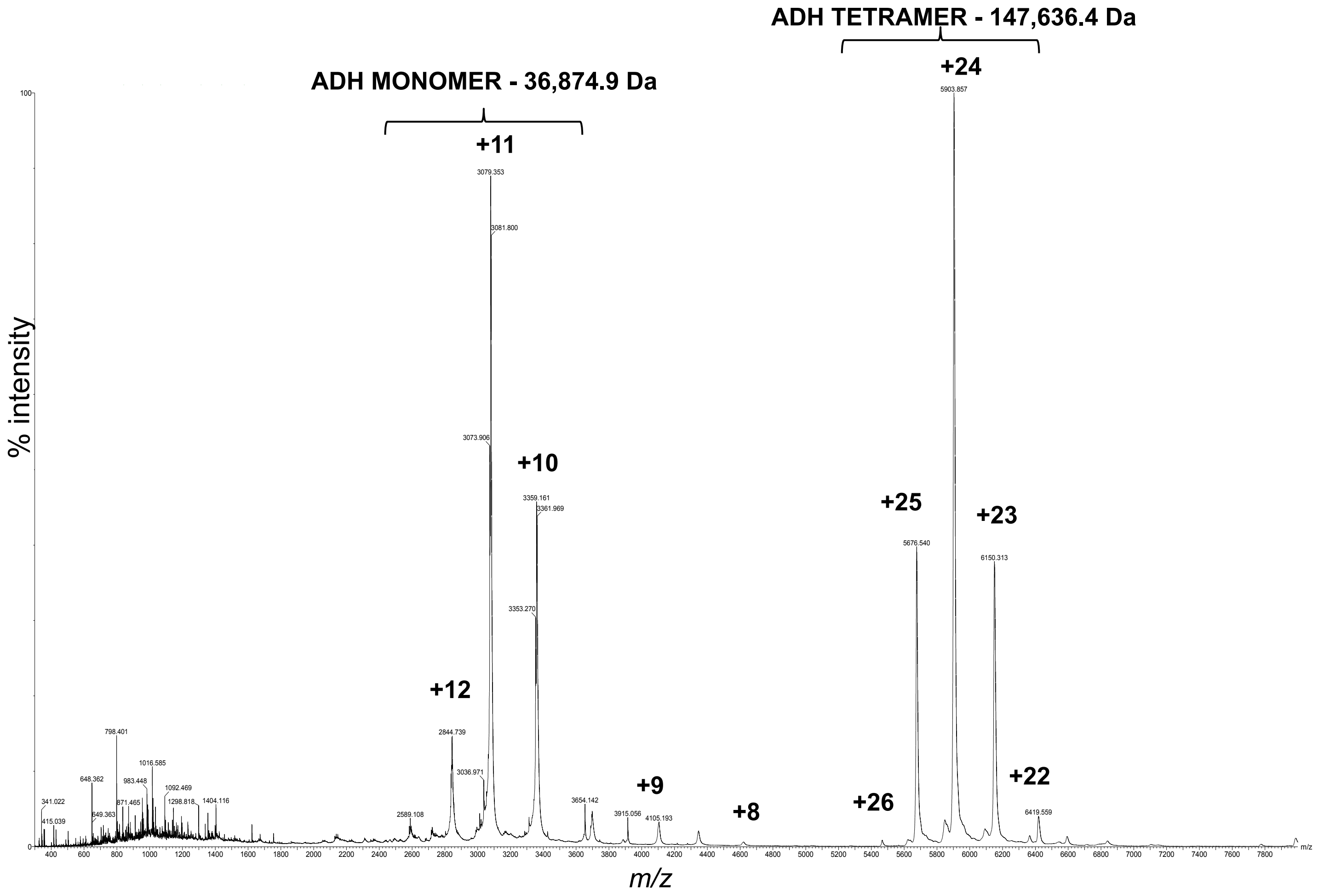
ADH TETRAMER - 147,636.4 Da
ADH MONOMER - 36,874.9 Da
% intensity
m/z
+24
+11
+10
+25
+23
+12
+22
+9
+8
+26
5903.857
3079.353
3081.800
3073.906
3359.161
3361.969
3353.270
5676.540
6150.313
2844.739
3036.971
2589.108
3654.142
3915.056
4105.193
6419.559
798.401
1016.585
648.362
983.448
1092.469
871.465
649.363
1298.818
1404.116
341.022
415.039

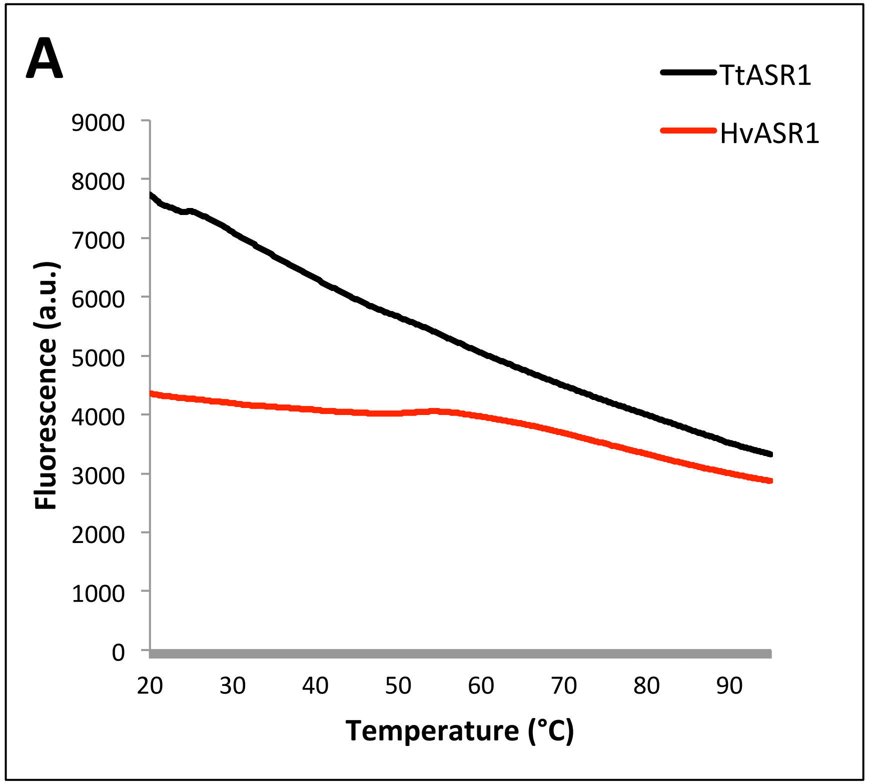

A
TtASR1
HvASR1
9000
8000
7000
6000
5000
4000
3000
2000
1000
0
Fluorescence (a.u.)
20
30
40
50
60
70
80
90
Temperature (°C)

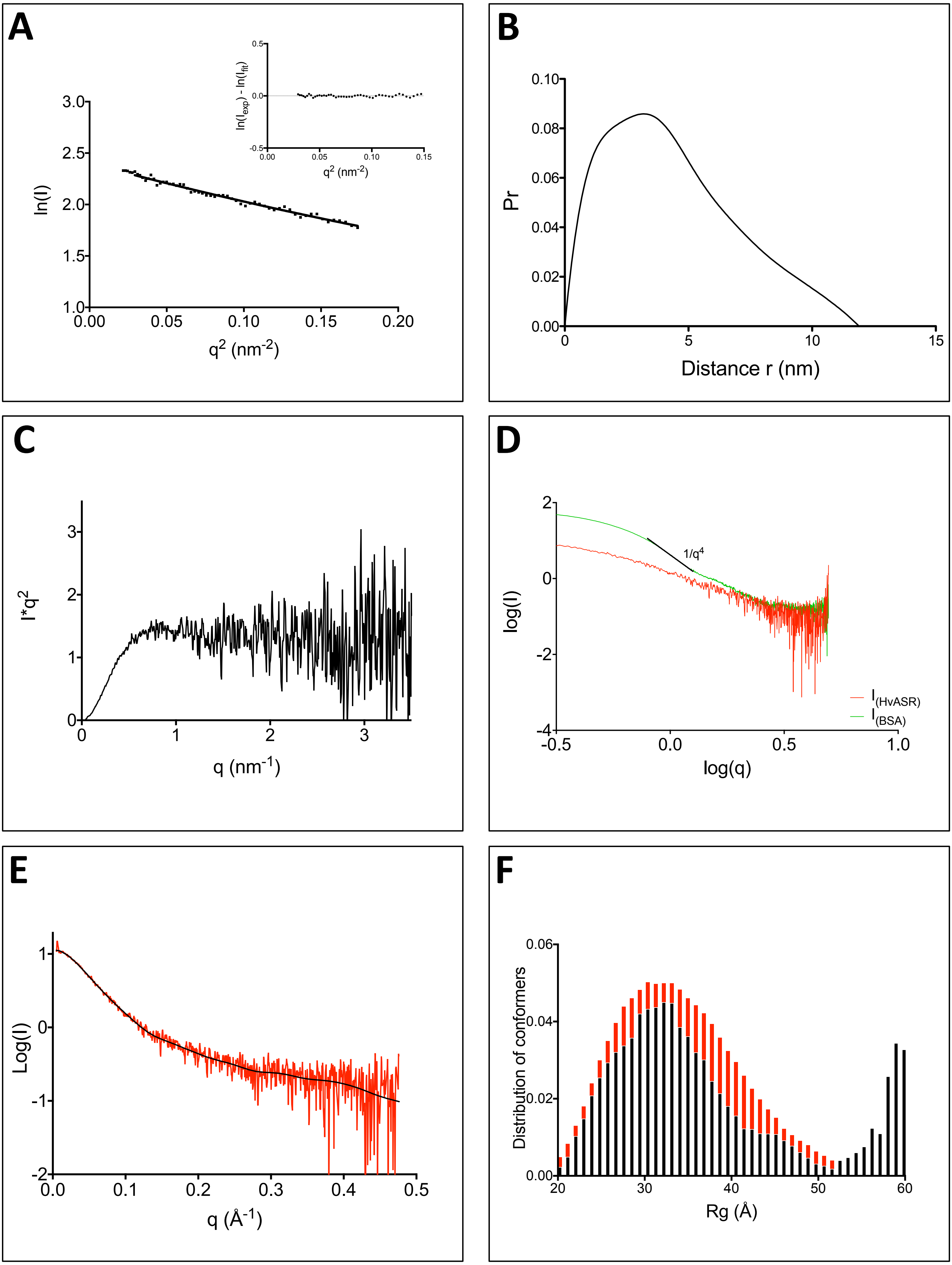
A
ln(I)
3.0
2.5
2.0
1.5
1.0
0.00
0.05
0.10
0.15
0.20
q2 (nm-2)
ln(Iexp) - ln(Ifit)
0.5
0.0
-0.5
B
Pr
0.10
0.08
0.06
0.04
0.02
0.00
0
5
10
15
Distance r (nm)
C
I*q2
q (nm-1)
D
log(I)
1/q4
I(HvASR)
I(BSA)
-0.5
0.0
0.5
1.0
log(q)
E
Log(I)
q (Å-1)
F
Distribution of conformers
0.06
0.04
0.02
0.00
20
30
40
50
60
Rg (Å)

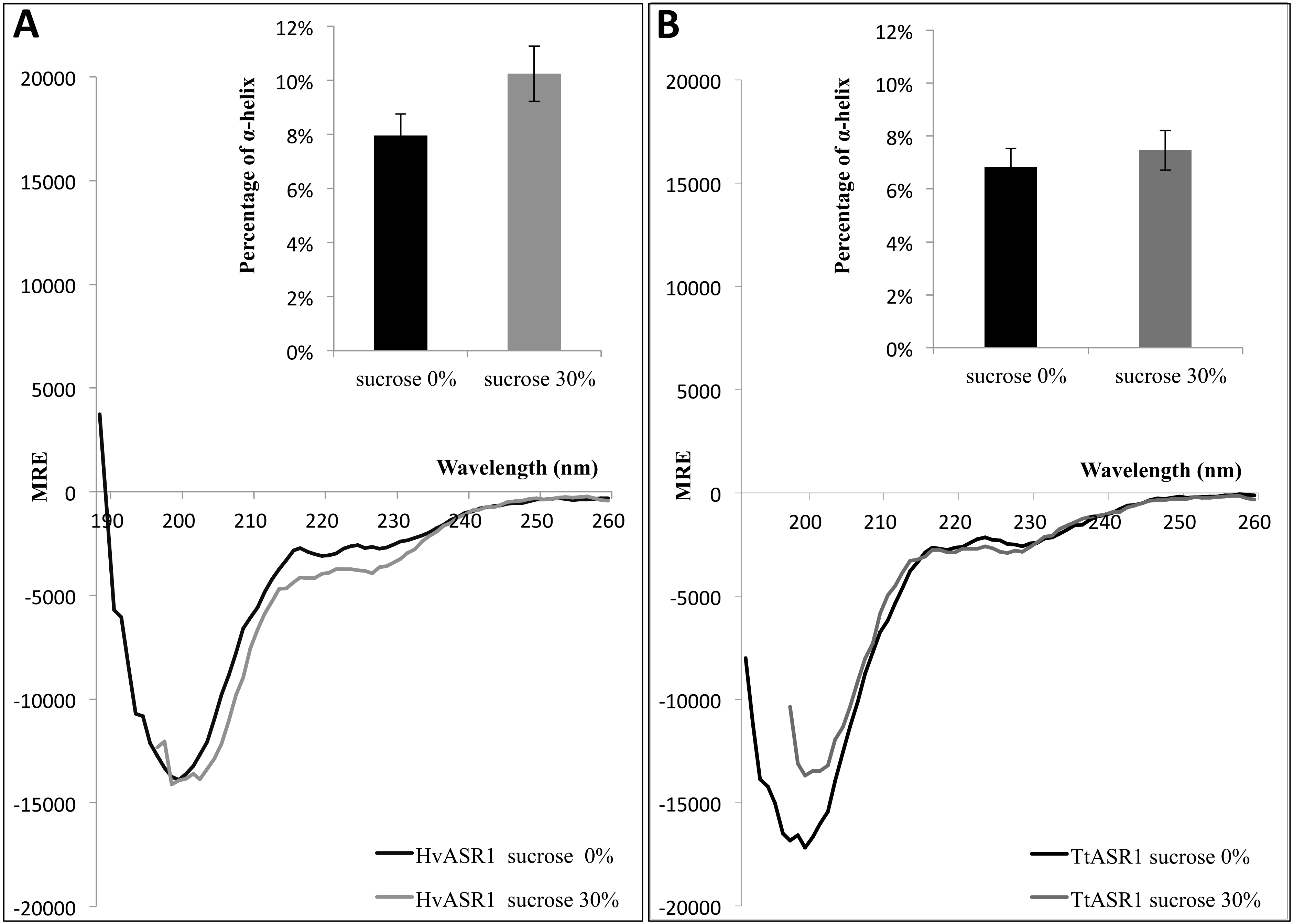

A
MRE
Wavelength (nm)
20000
15000
10000
5000
0
-5000
-10000
-15000
-20000
190
200
210
220
230
240
250
260
Percentage of α-helix
12%
10%
8%
6%
4%
2%
0%
sucrose 0%
sucrose 30%
HvASR1 sucrose 0%
HvASR1 sucrose 30%
B
MRE
Wavelength (nm)
20000
15000
10000
5000
0
-5000
-10000
-15000
-20000
200
210
220
230
240
250
260
Percentage of α-helix
12%
10%
8%
6%
4%
2%
0%
sucrose 0%
sucrose 30%
TtASR1 sucrose 0%
TtASR1 sucrose 30%

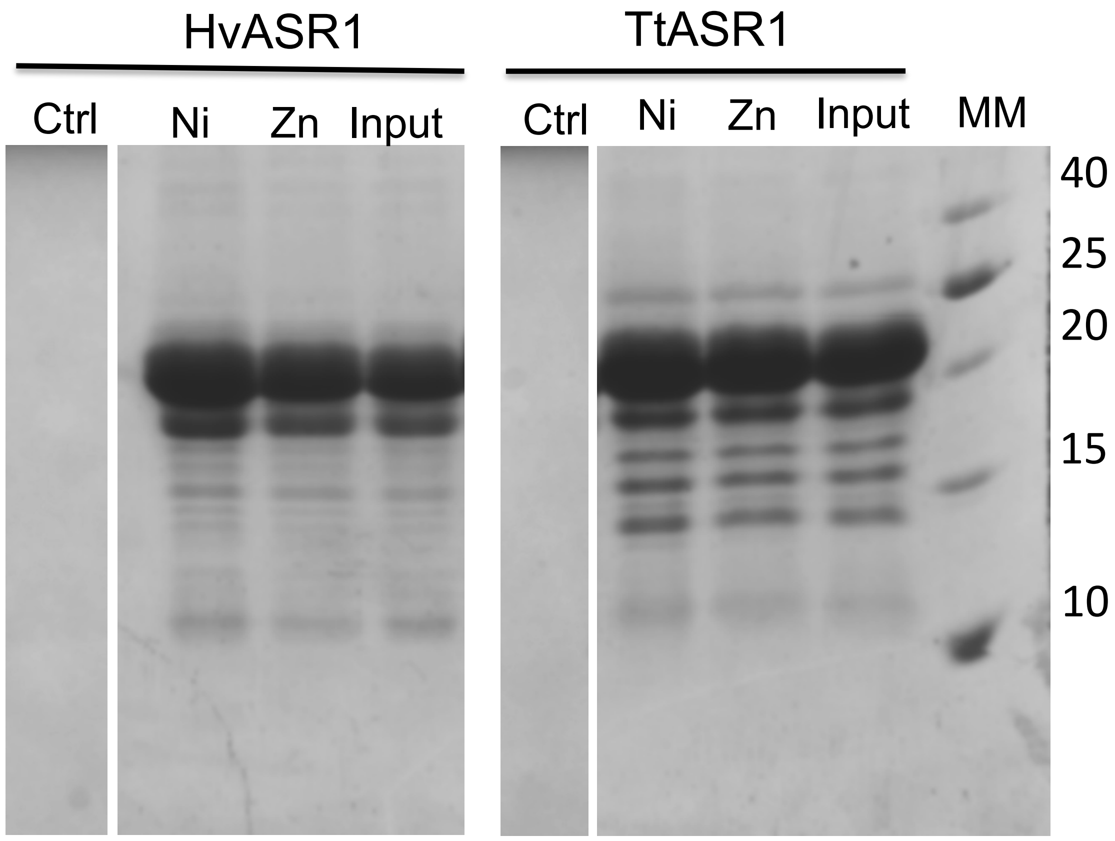
HvASR1
TtASR1
Ctrl
Ni
Zn
Input
Ctrl
Ni
Zn
Input
MM
40
25
20
15
10

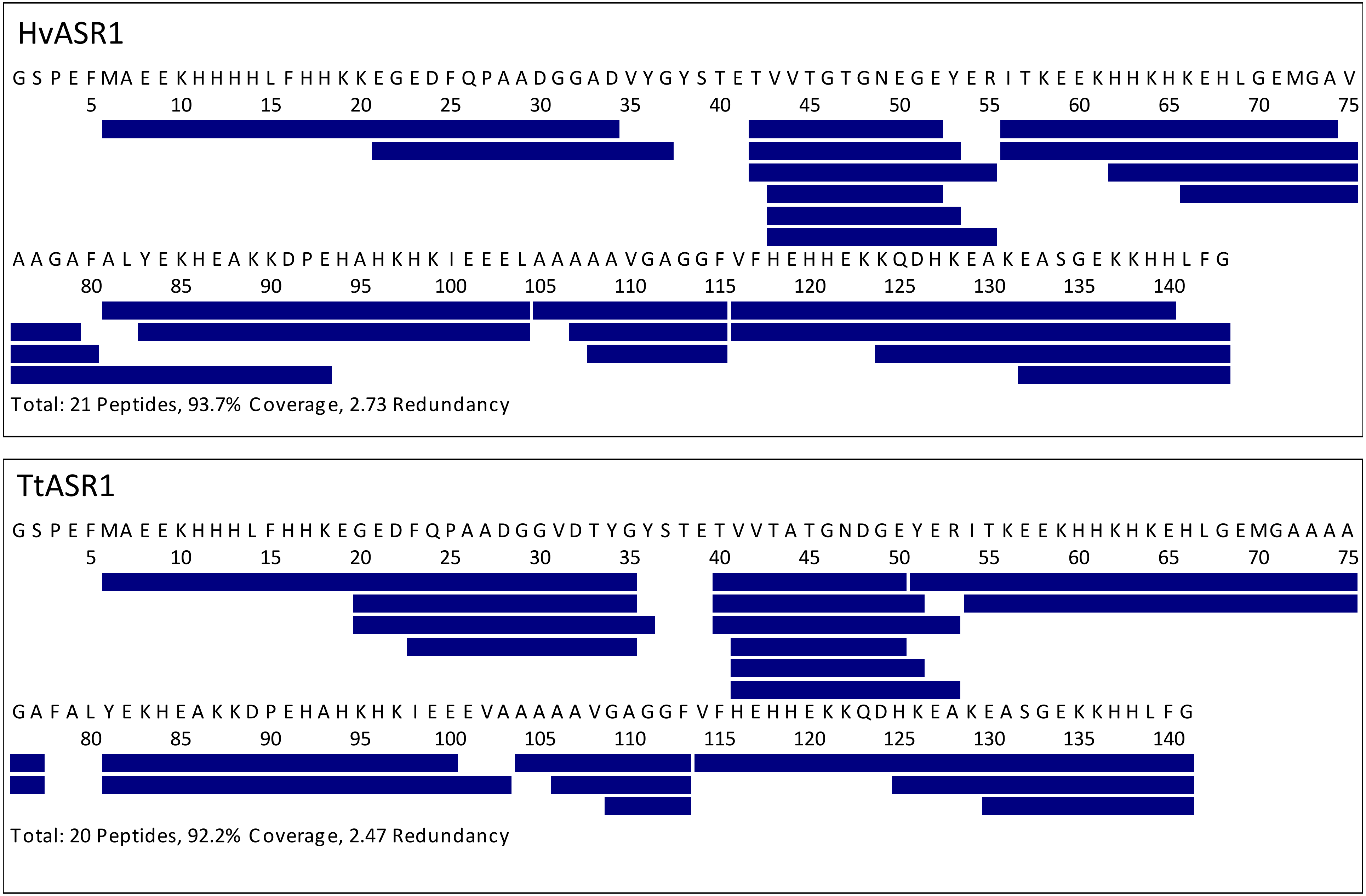

HvASR1
G S P E F M A E E K H H H H L F H H K K E G E D F Q P A A D G G A D V Y G Y S T E T V V T G T G N E G E Y E R I T K E E K H H K H K E H L G E M G A V
5 10 15 20 25 30 35 40 45 50 55 60 65 70 75
A A G A F A L Y E K H E A K K D P E H A H K H K I E E E L A A A A A V G A G G F V F H E H H E K K Q D H K E A K E A S G E K K H H L F G
80 85 90 95 100 105 110 115 120 125 130 135 140
Total: 21 Peptides, 93.7% Coverage, 2.73 Redundancy
TtASR1
G S P E F M A E E K H H H L F H H K E G E D F Q P A A D G G V D T Y G Y S T E T V V T A T G N D G E Y E R I T K E E K H H K H K E H L G E M G A A A A
5 10 15 20 25 30 35 40 45 50 55 60 65 70 75
G A F A L Y E K H E A K K D P E H A H K H K I E E E V A A A A A V G A G G F V F H E H H E K K Q D H K E A K E A S G E K K H H L F G
80 85 90 95 100 105 110 115 120 125 130 135 140
Total: 20 Peptides, 92.2% Coverage, 2.47 Redundancy
